# Supplementary material for: Realist evaluation of the role of the Universal Health Coverage Partnership in strengthening policy dialogue for health planning and financing: a protocol
Source: BMJ Open. 2019 Jan 17;9(1):e022345. doi: 10.1136/bmjopen-2018-022345 (PMC6340476; doi:10.1136/bmjopen-2018-022345)
Supplement: Author's manuscript [file bmjopen-2018-022345.draft_revisions.pdf]

BMJ Open is committed to open peer review. As part of this commitment we make the peer review history of every article we publish publicly available.

When an article is published we post the peer reviewers' comments and the authors' responses online. We also post the versions of the paper that were used during peer review. These are the versions that the peer review comments apply to.

The versions of the paper that follow are the versions that were submitted during the peer review process. They are not the versions of record or the final published versions. They should not be cited or distributed as the published version of this manuscript.

BMJ Open is an open access journal and the full, final, typeset and author-corrected version of record of the manuscript is available on our site with no access controls, subscription charges or pay-per-view fees (http://bmjopen.bmj.com).

If you have any questions on BMJ Open's open peer review process please email info.bmjopen@bmj.com

Realist evaluation of the role of the Universal Health Coverage Partnership in strengthening policy dialogue for health planning and financing: A protocol

Journal:	*BMJ Open*
Manuscript ID	bmjopen-2018-022345
Article Type:	Protocol
Date Submitted by the Author:	07-Mar-2018

Complete List of Authors:	Robert, Emilie; Research Institute of the McGill University Health Centre,<br>Ridde, Valery; Centre Population et Developpement; Universite de Montreal Institut de recherche en sante publique<br>Rajan, Dheepa; Organisation mondiale de la Sante, Department of Health Systems Governance and Financing<br>Sam, Omar; World Health Organization Inter-Country Support Team<br>Dravé, Mamadou; World Health Organization Country Office in Togo<br>Porignon, Denis; Organisation mondiale de la Sante, Department of Health Systems Governance and Financing

Keywords:	Health policy < HEALTH SERVICES ADMINISTRATION & MANAGEMENT, realist evaluation, policy dialogue, universal health coverage, health planning, health financing

Note: The following files were submitted by the author for peer review, but cannot be converted to PDF. You must view these files (e.g. movies) online.

Note from the Editors for study protocols.docx

SCHOLARONE™ Manuscripts

TITLE PAGE

Title of the article

Realist evaluation of the role of the Universal Health Coverage Partnership in strengthening policy dialogue for health planning and financing: A protocol

Full name, postal address, e-mail and telephone number of the corresponding author

Full name : Emilie Robert

Postal address : Institut de recherche du Centre universitaire de santé McGill

2155, rue Guy, 5e étage

Montréal (QC)

Canada H3H 2R9

E-mail : emilie.robert2@mail.mcgill.ca

Telephone number : +1.514.998.9892

Full name, department, institution, city and country of all co-authors

- Valéry Ridde

  French Institute For Research on sustainable Development
  CEPED (IRD-Université Paris Descartes), Universités Paris Sorbonne Cités, ERL INSERM SAGESUD
  Paris, France

  AND

  University of Montreal Public Health Research Institute (IRSPUM)
  Montreal, Canada

- Dheepa Rajan

  Department of Health Systems Governance and Financing
  World Health Organization
  Geneva, Switzerland

- Omar Sam

  Inter-Country Support Team
  World Health Organization
  Ouagadougou, Burkina Faso

For peer review only

- Mamadou Dravé (at the time of the pilot mission)

  World Health Organization Office in Togo
  Lomé, Togo

- Denis Porignon

  Department of Health Systems Governance and Financing
  World Health Organization
  Geneva, Switzerland

Word count, excluding title page, abstract, references, figures and tables

4,216

For peer review only

ABSTRACT

INTRODUCTION

In 2011, the World Health Organization (WHO), the European Union and Luxembourg entered into a collaborative agreement to support policy dialogue for health planning and financing; these were acknowledged as core areas in need of targeted support in countries' quest towards universal health coverage (UHC). Entitled 'Universal Health Coverage Partnership', this intervention is intended to strengthen countries' capacity to develop, negotiate, implement, monitor, and evaluate robust and integrated national health policies oriented towards UHC. It is a complex intervention involving a multitude of actors working on a great number of extremely diverse activities in different countries.

METHODS AND ANALYSIS

The researchers will conduct a realist evaluation to answer the following question: How, in what contexts, and triggering what mechanisms, does the Partnership support policy dialogue for health planning and financing towards UHC? A qualitative multiple case study will be undertaken in Togo, Liberia, Democratic Republic of Congo, Cape Verde, Burkina Faso and Niger. Three steps will be implemented: 1) reviewing the literature to inform reflection on the intervention theory and to suggest propositions that will guide the data collection; 2) collecting empirical data through semi-structured interviews with key informants (e.g. WHO, international organizations, Ministries of Health, etc.), observations of key events; documentation; and feedback sessions with WHO; 3) testing the propositions on empirical data to fine-tune the intervention theory.

ETHICS AND DISSEMINATION

The main target audiences are WHO and its partner countries; international and national stakeholders involved in or supporting policy dialogues in the health sector, especially in low-income countries; and researchers with interest in UHC, policy dialogue, evaluation research, and / or realist evaluation. WHO AFRO ethics review committee approved the study (AFR/ERC/2017/02.08). WHO as the funder of this research will not have any role in data collection and data analysis. However, any publication will require WHO approval.

KEY WORDS

Heath policy; realist evaluation; policy dialogue; universal health coverage; health planning; health financing

For peer review only

STRENGTHS AND LIMITATIONS OF THIS STUDY

1. The internal validity is ensured by the in-depth study of six countries; and the potential to generalize the findings are increased by building explanations, taking the context into account in the production of outcomes, and using existing literature to provide theoretical foundations to the findings.
2. Methodological developments are expected, as the study is among the few realist evaluations to focus on policy dialogue, especially in low- and middle-income countries.
3. The study involves training and ongoing supervision of West African researchers who come from a different educational background, contributing to capacity building in health policy and systems research in low- and middle-income countries.
4. The study does not include countries or cases outside of the West African region.
5. Comprehensive data collection may be challenging in some countries with an unstable political situation.

For peer review only

INTRODUCTION

Universal health coverage (UHC) as a core objective within the Sustainable Development Goals is a journey in which multiple stakeholders, from the local, national and international levels, partake. It involves on-going discussions and negotiations among these stakeholders on the different facets of UHC, among which financing and planning questions.[1, 2] Under the leadership of national health authorities, stakeholders will thus have to agree on priorities for action, and health financing and health system organization modalities.

Policy dialogue is where such discussions take place. The World Health Organization (WHO) describes policy dialogue as an iterative process that targets both the technical and policy aspects of the problem being discussed, involving evidence and sensitive policy discussions, in which a wide range of stakeholders participate.[3] This dialogue has a concrete objective, such as the development of a plan, a strategy or a policy. Policy dialogue is thus understood as a deliberative process by which different stakeholders are brought together to discuss issues of public policy to feed into decision-making.

In low- and middle-income country (LMIC) settings, the topic area of policy dialogue is attracting interest as a target intervention for programmes. Policy dialogue is seen as a key governance tool to ensure the coordination and alignment of stakeholders and to develop robust and comprehensive health policies anchored in the UHC concept.[4] Negotiations in the health sector may indeed be challenging due to the heavy influence external aid can have on national priorities, combined with the often limited government steering capacity to counter-balance that influence.[5, 6] In the context of UHC, Ministries of Health's convening and brokering role takes on an added layer of importance, as they need to bring together a diverse range of interests, and at times, conflicts of interests, to mobilize resources, prioritize interventions, and strengthen institutions for UHC.

Following this trend, policy dialogue is becoming a popular research object.[7] In the field of health policy and systems research in LMICs, scholars study policy dialogue as a knowledge translation instrument,[8-11] or as a decision-making process. In this case, they notably study the perceptions of what policy dialogue is.[12-14] The actual process of policy dialogue is more rarely investigated. [15, 16] In high-income countries, research on policy dialogue as part of decision-making is more diffuse and includes research on research-policy dialogue,[17, 18] stakeholder dialogue,[19, 20] or social dialogue.[21]

In 2011, WHO, the European Union and Luxembourg entered into a collaborative agreement called the *Universal Health Coverage Partnership* to support policy dialogue for health planning and financing as core topic areas of UHC. This Partnership also responds to a resolution from the 64th World Health Assembly calling on WHO to support countries in developing more robust and evidence-based health policies and plans based on an inclusive and participatory process.[22] Implemented in 28 countries, the Partnership is a complex intervention involving a multitude of actors working on a great number of very diverse activities in different countries. Moreover it takes place in the policy arena where policy making is complex and dynamic. This study aims to examine the Partnership through a realist lens, to better understand if it can, through WHO in-country support, contribute to strengthening policy dialogue, how and in what contexts.

For peer review only - http://bmjopen.bmj.com/site/about/guidelines.xhtml

REALIST APPROACH

Traditional evaluation methods face three major limitations when studying complex interventions in dynamic contexts, as is the case of the Partnership. First, establishing a causal relationship between the Partnership and its expected outcomes in terms of policy formulation and donors alignment on one hand, and between the Partnership and the indicators normally used in classical evaluations on the other, is onerous as such relationships are not direct and strongly colored by the contexts within which WHO intervenes. Second, WHO's role in the Partnership complements its traditional role as technical expert. Demonstrating how this new approach helps support policy dialogue, and painting a clear picture of the challenges involved, is difficult to do using traditional evaluation methods. Finally, the Partnership has been implemented in several countries representing a wide variety of contexts, which raises the third limitation of traditional evaluations: the inability to take context into account in explaining an intervention's outcomes and to draw parallels between the experiences of different countries. Therefore, the realist approach was deemed most appropriate as a methodology to evaluate the UHC Partnership.

Realist evaluation of interventions was popularized by Pawson and Tilley.[23] Rooted in critical realism, it understands causality as being generative, which entails the premise that an intervention does not function on its own and is not, in itself, what produces an outcome. Rather, an outcome is the product of the interaction between a mechanism triggered by the intervention and the context in which the intervention is implemented. A mechanism should be understood as the way in which the actors involved in an intervention react to and reason about the resources made available to them as part of the intervention. Table 1 presents the three broad characteristics of a mechanism, as described by Lacouture and colleagues. [24]

Table 1. The characteristics of mechanisms according to the realist approach, by Lacouture et al. [24]

**A mechanism is hidden but real.**<br><br>Already present before the intervention, but latent, a mechanism reveals itself over the course of an intervention's implementation in a given context. Sensitive to the context, it produces outcomes.
**A mechanism is an element of reasoning or of an agent's reaction…**<br><br>… given his/her capacity to use the resources made available as part of an intervention, in a certain context, in order to effect change.
**A mechanism evolves in open space–time and in a system of open relationships.**<br><br>It is in interaction with other mechanisms, with the context, and with the outcomes that it has itself produced (feedback loop).

A realist evaluation therefore does not attempt to control the variables associated with the implementation context. Rather, it seeks to observe, in the natural environment, the regular—although not systematic—occurrence of an outcome in a particular context, when a mechanism is triggered. These iterations of interactions are referred to as context–mechanism–outcome (CMO) configurations. The objective of a realist evaluation is thus to identify, in the empirical data, iterative interactions between an intervention's context, mechanisms, and outcomes.

Realist evaluation is rooted in the theory-based evaluation movement.[25] In essence, interventions are theories: they are based on the idea that an intervention is expected to produce

For peer review only - http://bmjopen.bmj.com/site/about/guidelines.xhtml

an outcome for various reasons related to the activities implemented.[26] Operationally, a realist evaluation begins with a reflection on the intervention theory and results in the production of an explanatory theory, which is sufficiently abstract to explain how the intervention functions in different contexts. This anchoring in theory requires an explicitly iterative process between the conceptual and theoretical literature underlying the rationale behind the intervention, and the empirical data.

INTERVENTION THEORY

Three sources of information were used to develop an initial realist intervention theory of the Partnership. In addition to reviewing the documents produced by WHO, several meetings were held with stakeholders from WHO headquarters' Department of Health Systems Governance and Financing which implements the Partnership. These meetings aimed to make explicit the logical reasoning underlying the need for and the rationale behind the Partnership. Observations of two annual inter-country meetings bringing together partner countries and WHO teams were also conducted. Finally, semi-structured interviews were conducted with stakeholders at WHO headquarters and at regional level. The scientific literature on policy dialogue was also examined to highlight mechanisms at play. This process resulted in a generic intervention theory that was discussed at a workshop in Burkina Faso in 2017, and subsequently adjusted to take into account participants' input (Figure 1).

In the frame of the Partnership, WHO supports policy dialogue processes for health planning and health financing. According to WHO, such processes should be inclusive and participatory, fed by knowledge and evidence, and led by the Ministries of Health (MoH). WHO's role is to support MoH to lead structured and transparent policy dialogues. The ultimate goal is to contribute to strengthening health systems, especially its governance function, with the assumption that health systems strengthening is the principal means to achieve UHC. WHO support through the Partnership manifests itself through a hard component, including financial resources for a set of activities as well as human resources, and a soft component, which refers to a specific approach to supporting the policy dialogue. The hard component of WHO support includes:

1) Strengthening of the capacities of MoH to conduct policy dialogue processes; this involves WHO country office on-going support as well as training;

2) Strengthening of the quality of the policy dialogue itself with the generation of relevant evidence, data and normative guidelines, as well as *ad hoc* expertise.

With regard to WHO's approach, three characteristics are key: flexibility, i.e. the ability to adapt to the changing priorities of the MoH; responsiveness, or the ability to position and respond to requests from the MoH; and proactivity, i.e. the ability to anticipate implementation steps and expectations or needs of the MoH.

The resulting policy dialogue is expected to enable the formulation of robust and comprehensive health policies, strategies and plans that promote the principles of UHC such as equity, financial risk protection, and quality of care. It should also facilitate the alignment of multiple stakeholders, be they donors, civil society, or related ministries. Thus, the Partnership is

For peer review only - http://bmjopen.bmj.com/site/about/guidelines.xhtml

supposed to contribute to upholding the principles of the Paris Declaration to ensure effective development cooperation. Finally, supporting MoH in the organization of such dialogue is expected to strengthen the capacities and leadership position of the MoH. To summarize, WHO has three main roles within the Partnership: convener of policy dialogue processes; knowledge broker; and a relatively traditional role of technical advisor to the MoH.

Within a realist perspective, researchers raise the question of the mechanisms, that is, how agents react to the new resources made available through an intervention. This concerns first the beneficiaries of the Partnership, including the MoH and all stakeholders involved in the policy dialogue. Thus an inclusive, participatory and knowledge-driven policy dialogue should first trigger a shared understanding of stakes, values and interests of the various actors involved,[19, 27, 28] as well as of the knowledge and possible ways of action to try to overcome the challenges.[17, 19]. Then, policy dialogue should prompt stakeholders' trust in a structured and transparent process and buy-in of the decisions resulting from the dialogue,[19] strengthening their alignment on national health priorities. Finally, brought under the responsibility of MoH supported by WHO, such a policy dialogue should generate a sense of MoH ownership of such processes, contributing to the strengthening of its leadership in the health sector.

Although the Partnership emphasizes the policy dialogue processes and their outcomes, its resources are primarily targeted at the actors in charge of supporting the policy dialogue. These actors are not only MoH staff who benefit from WHO training and support, but also the Partnership focal points in WHO country offices. The latter are either international experts mobilized on a full-time basis in some countries, or responsible for two countries at once, or national experts in health system strengthening. They are the implementers of the Partnership. Therefore mechanisms triggered by their reactions and reasoning should also be investigated. However these mechanisms remain to be uncovered at the time of the protocol.

(Insert Figure 1)

METHODS AND ANALYSIS

RESEARCH QUESTIONS

The question that will guide the study is: How, in what contexts, and triggering what mechanisms does the Partnership support policy dialogue for health planning and health financing towards UHC?

To respond to this research question, four specific objectives have been established:

1) To highlight the contexts in which the Partnership, through WHO support, can, or cannot:

- act as a broker and a convener, creating synergy among the actors involved in policy dialogue for health planning and financing;
- play its role as technical expert, ensuring transparent and structured policy dialogue in accordance with available evidence and equity principles;

For peer review only

- support MoH in its leadership and stewardship functions.

2) To identify, for each of these mandates, the mechanisms at work and the outcomes—direct or indirect, expected or not—produced;

3) To clarify the process chains and the links between them, especially highlighting feedback loops;

4) To propose a theory explaining how, in what contexts, and triggering what mechanisms the Partnership, through WHO support, enhances policy dialogue for health planning and financing towards UHC.

STUDY DESIGN

A realist multiple case study will be undertaken.[29] Table 2 presents a summary of the proposed design. The countries in which the study will be conducted are Togo, Liberia, Democratic Republic of Congo, Cape Verde, Burkina Faso and Niger. Due to feasibility concerns, only partner countries of the African continent were selected. They were sampled using a non-probabilistic approach. The objective was to select cases—or countries—purposively, so as to obtain a diversity and depth of information on the Partnership. This is therefore a contrasted sample. When selecting cases, purposive sampling involves establishing selection criteria that ensure contrasting cases. The criteria are based on theoretical assumptions about the influence they may have on the Partnership and policy dialogue. An initial list was shared with WHO in order to select the criteria that could be informed by their experience. Based on their country knowledge and experience, they then suggested countries matching the criteria, based on their country knowledge and experience (Table 3).

Table 2. Summary of the study design

**Epistemological foundation**	Critical realism
**Design**	Multiple case study
**Cases**	Countries involved in the Partnership
**Unit of analysis**	Processes implemented as part of the country roadmaps
**Type of sample**	Contrasted
**Case sampling strategy**	Purposive sampling

For peer review only - http://bmjopen.bmj.com/site/about/guidelines.xhtml

Table 3. Case selection criteria and reason for sampling

Criteria*	Togo	Liberia	DRC	Cape Verde	Burkina Faso	Niger
Duration of WHO support	4	4	3	3	3	3
Political stability (0=instable; 3=stable)	3	3	2	3	3	1,5
Ministerial stability (0=instable; 3=stable)	3	3	3	2	2	1
Openness to policy dialogue (0=not open; 3=open)	3	3	3	2	2	1,5
Ministry of Health leadership capacity (0=weak; 3=strong)	3	3	3	3	3	1,5
Implementation of the national road map (0=weak; 3=good)	3	3	3	2	3	1
Presence of entry points	Yes	Yes	Yes	Yes	Yes	Yes
Additional observations		*Ebola epidemic*	*Devolution*	*Archipelago – Lower middle-income country*		

* *At the time of case selection*

Each country has a roadmap for UHC Partnership work, in line with its needs. In this study, a unit of analysis is a policy dialogue process defined in the country's roadmap. One process at least (unit of analysis) will be observed in each country (case). Each process was selected in collaboration with the country teams, taking into account on-going support at the start of data collection, as well as opportunities for observation. In Togo and DRC, the processes under study are the policy dialogues around the national health financing strategy, whereas in Cape Verde, Niger and Togo, there are the policy dialogues for the elaboration of the national or regional health plans. In Liberia, the process under study is the elaboration of the IHP+ (International Health Partnership) Compact, which is an aid coordination agreement amongst health sector stakeholders. The process selected determines the research sub-questions to be addressed in each case, the documentation to be obtained, the informants to interview, and the topics to be covered in interviews.

STUDY STEPS

Step 1: Suggesting theoretical propositions

An initial theory, considered as the starting point, offers an explanation for the participative and inclusive approach adopted by WHO in the policy dialogue: the theory of partnership synergy. According to this theory, WHO would opt for a participatory and inclusive approach on the premise that collaboration improves the capacity of both individuals and organizations to achieve their objectives thanks to the partnership synergy mechanism. Synergy is "the power to combine the perspectives, resources, and skills of a group of people and organizations."[30] This

For peer review only - http://bmjopen.bmj.com/site/about/guidelines.xhtml

collective approach generates actions and ideas that are new and relevant, contributing more to the achievement of the objectives than the sum of individual contributions would have done.

In that context, WHO assumes the role of "boundary spanner", i.e., an actor who brings together partners with common interests and goals (such as UHC), while striving to establish a climate of trust, optimism, and perseverance.[31] Inspired by Jagosh and colleagues,[32] three initial theoretical propositions were developed:

- The current upstream context of the Partnership influences the degree to which WHO can promote collaboration among the many stakeholders.
- The synergy generated by collaboration among stakeholders in the Partnership advance the policy dialogue farther than could be accomplished by the individual contributions of each stakeholder.
- The synergy generated in the context of the Partnership has cumulative effects (e.g. improved climate of trust, shared understanding of health challenges) over time. This means, for example, that an outcome produced by policy dialogue at one stage of the Partnership will influence later stages (feedback loops).

The concept of policy broker is similar to that of boundary spanner. The role of a broker is to bring together groups of actors whose interests and beliefs differ with regard to the issue of concern in the policy dialogue.[33] The broker's objective is to promote consensus in a climate of trust, in order to stabilize the policy dialogue and to find workable compromises. However, his role is not necessarily a disinterested one, and he might be pursuing a strategic objective, which would bring him closer to the role of policy entrepreneur. A policy entrepreneur has an explicit strategic objective, and proposes ideas and stimulates opportunities in ways that are intended to create a demand for the solution he is proposing.[33]

These roles are, however, ideal types positioned at two ends of a continuum.[33] Depending upon the contexts in which it intervenes, the processes in which it is involved, and the issues being discussed, WHO may be called upon to play one or another of these various roles, deploying different types of mechanisms, beyond synergy, and thereby influencing the outcomes of the Partnership. Consequently, further theoretical propositions will be proposed in later stages of the study, following a review of the conceptual and theoretical literature.

Step 2: Collecting empirical data to test the theoretical propositions

Three sources of information will be used. First, semi-structured interviews will be conducted with key informants who will be jointly identified by the researchers and WHO. An initial list will be compiled by WHO and then supplemented using a snowballing technique. The aim is to meet with as many as possible of the participants of the policy dialogue and avoid investigating merely the positive side of the coin, and thus possible bias. Table 4 presents the possible—and non-restrictive—categories of informants. Depending on informants' availability, interviews will be conducted in person or by telephone. The interviews will be audio-recorded, except when not allowed by the participants. They will also be recorded in notes taken systematically by the researchers. Recordings will subsequently be transcribed. Between 20 and 30 informants will be interviewed for each case. The principle of empirical saturation will be applied to determine the

For peer review only - http://bmjopen.bmj.com/site/about/guidelines.xhtml

best time to bring the data collection to a close.[34] For each interview, a list will be prepared of topics to be addressed and of clarifications to be made regarding their understanding and knowledge of the processes. This list will guide the interview and will focus on the following elements of information:

- understanding the policy dialogue processes;
- highlighting the roles, visions, and objectives of the various stakeholders involved in the policy dialogue;
- describing the successes and challenges of these processes.

Table 4. Distribution of key informants

World Health Organization	• headquarters team<br>• regional team<br>• country teams
Ministry of health, connected ministries, and public institutions	• Ministry managers<br>• others to be determined
Actors in international institutions and civil society involved in policy dialogue and health planning	• *ad hoc*, to be determined

Observations will also be conducted of activities organized either directly by WHO or with WHO collaboration within the Partnership context. These observations will be described and recorded in the logbooks of researchers. Also, all documentation related to the Partnership (e.g. national roadmaps, project documents, annual reports) will be reviewed.

Furthermore, to gain a deeper understanding of the national health policy contexts, and particularly of the interests, power relationships, and positioning of the actors involved in policy dialogue, a stakeholder analysis will be performed for each case.[35] First, we will compile a list of stakeholders involved in policy dialogue for each country. Then, for each stakeholder, we will attempt to describe their power of action and influence, as well as their interest and involvement with respect to UHC and policy dialogue. The last step will be to analyze how these stakeholders react to the roles played by WHO in policy dialogue, and how their reactions influence these two processes. This exercise will be conducted before the interviews in collaboration with the WHO teams, who have first-hand knowledge of the field. The analysis will be completed during the interviews with the stakeholders included on the list.

Step 3: Testing the theoretical propositions and specifying the theory

CMO configurations will be identified in the data through content analysis performed using NVivo software, according to the following steps: 1) identify the outcomes (description); 2) identify the contextual components related to the outcomes (resolution); 3) reconstruct

For peer review only - http://bmjopen.bmj.com/site/about/guidelines.xhtml

theoretically the links between context and outcomes (abduction); and 4) identify the mechanisms (retroduction).[36] Retroduction aims to construct the theoretical explanation of a phenomenon.[37, 38] More specifically, this retrospective reasoning allows to move from describing the outcome to describing what produced that outcome and the context within which it takes place,[39, 40] via the mechanisms.[41] The configurations are most often incomplete, with the mechanism generally not being explicit. The missing links will thus be inferred from the theoretical literature, which provides clues to explain the links between causes and outcomes, following a process of intellectual reasoning based on two questions: How can we explain the mechanism in this context and considering this outcome? Is this interpretation supported by the theoretical literature?

This inference is the first level of abstraction and takes the form of a semi-regularity when the configuration re-appears several times. A semi-regularity is a regular—although not systematic—occurrence in the interactions among the context, the mechanism(s), and the outcomes.[42] When a configuration is identified only once but its interpretation is supported by the theoretical literature, it is also taken into account, on the condition that it helps to clarify WHO's role. Each semi-regularity will provide one or more piece of information that, when combined, present a more complete and precise picture of WHO's role. Such an analysis will contribute to specify the explanatory realist theory of the Partnership.

ETHICS AND DISSEMINATION

PROTECTION OF KEY INFORMANTS

The research objectives and a consent form will be sent by email to informants before the interviews. The rights of informants include the right to refuse to participate in the interview, to end their participation in the study at any time, and to not allow their statements to be audio-recorded. Informed consent will be sought in writing from informants. We expect that all informants will be literate, given that they occupy positions of responsibility.

The main risk associated with the study is the impact that informants' statements in interviews could have on their relationships with their partners, including their employers, collaborators or actors with whom they are involved in common activities, to the extent that these statements might be perceived as offensive. Consequently, informants' statements will be kept confidential. The data collected will be kept anonymous at the time of transcription, i.e., informants' names will be concealed, as well as their title and their function. Transcriptionists and everyone involved in data collection and analysis will sign confidentiality agreements. Transcripts and recordings will be available only to the researchers and those performing data analysis. A copy of the data will be maintained on a password-protected virtual platform. If there is any risk that participants could be identified from their statements, researchers will reinforce their anonymity, for example, by not citing the statements directly or by not indicating any association with their employer. The data will be destroyed 18 months after the signing of the consent forms.

ETHICS APPROVAL OF RESEARCH

The research protocol has been approved by the Ethics Committee of the WHO Regional office for Africa (ARF/ERC/2017/02.08), as well as the Ethics Committee of Burkina Faso (2017-01-

For peer review only - http://bmjopen.bmj.com/site/about/guidelines.xhtml

007), Cape Verde (CAV/WR/402/2016), Liberia (17-03-031), Togo (007/2017/CBRS) and DRC (038/CNES/BN/PMMF/2017). Approval of Ethics Committee in Niger is pending.

IMPORTANCE OF THE STUDY

First, in providing lessons on how the Partnership works and what proximal and unexpected outcomes it produces, the research should allow WHO and Partnership donors to improve the Partnership content, to adapt it better to the contexts in which it is implemented, and to tailor the nature of the policy dialogue support to take into account the diverse influences that foster or undermine productive collaboration among stakeholders. This more constructive collaboration should in turn contribute to more robust and actionable health policies, as a means to strengthen health systems so that they can deliver the quality and affordable health services users are entitled to under the banner of UHC. Consequently, the primary beneficiaries of this study are the WHO, as the implementer of the Partnership, as well as its donors. In addition, key beneficiaries are the different Partnership country MoHs, one of the main targets of WHO support. The secondary beneficiaries are the international and local stakeholders who participate in the policy dialogues supported by the WHO. Users of health services and the population in general are indirect beneficiaries, provided that the WHO and donors take the findings of the research into consideration.

Second, the realist evaluation has been little used to study interventions on an international scale. Thus, this study will be a unique example in health policy and systems research, and will offer important methodological lessons. Furthermore, it entails the training and ongoing supervision of a team of West African researchers coming from various educational backgrounds and with different expertise. This project will help strengthen capacities in health policy and systems research in low- and middle-income countries.

Finally, in terms of the topics addressed, the nature of the knowledge produced will be unique and will provide a deeper understanding of the issues associated with interventions aimed to support policy dialogue. As such, it adds to the findings on policy dialogue reported in the *BMC Health Services Research* special issue.[43]

DISSEMINATION OF RESULTS

The results of the study will be disseminated on several levels: WHO, including the headquarters, the regional teams, and the country teams; WHO's national and international partners, particularly MoH and donors; and the scientific community. Presentation workshops with the country teams will be organized at the end of each data collection to present preliminary results. A final presentation workshop will be organized when all the data have been collected and analyzed, to inform WHO of the findings.

With regards to the scientific community, empirical findings and methodological issues will be shared in related symposia and in peer-reviewed journals. Any intention to publish the findings must be approved by WHO before submitting to the journal.

For peer review only - http://bmjopen.bmj.com/site/about/guidelines.xhtml

REFERENCES

1 Schmets G, Rajan D, Kadandale S. *Strategizing national health in the 21st century: a handbook*. Geneva: World Health Organization, 2016.

2 Evans T, Pablos-Mendez A. Shaping of a new era for health financing. *Lancet* 2016;**387**(10037):2482-4 doi: 10.1016/S0140-6736(16)30238-0.

3 World Health Organization. Health Systems Strengthening: Glossary. Geneva, Switzerland: World Health Organization, 2011.

4 Rajan D, Adam T, El Husseiny D, et al. Briefing Note - Policy dialogue: What it is and how it can contribute to evidence-informed decision-making. Gevena: WHO, 2015.

5 Uneke CJ, Ezeoha AE, Ndukwe CD, et al. Enhancing leadership and governance competencies to strengthen health systems in Nigeria: assessment of organizational human resources development. *Healthc Policy* 2012;**7**(3):73-84

6 Quinn M. Governance and Health in Post-Conflict Countries: The Ebola Outbreak in Liberia and Sierra Leone. New York: International Peace Institute, 2016.

7 Nabyonga-Orem J, Ota M, Mwisongo A, et al, eds. Health policy dialogue: lessons from Africa. *BMC Health Serv Res* 2016;**16**(Supplement 4)

8 Lavis JN, Boyko J, Oxman AD, et al. SUPPORT Tools for evidence-informed health Policymaking (STP) 14: Organising and using policy dialogues to support evidence-informed policymaking. *Health Res Policy Syst* 2010;**10**(5):514-19 doi: 10.1186/1478-4505-7-S1-S14.

9 Uneke CJ, Ebeh Ezeoha A, Uro-Chukwu H, et al. Promoting Evidence to Policy Link on the Control of Infectious Diseases of Poverty in Nigeria: Outcome of A Multi-Stakeholders Policy Dialogue. *Health Promot Perspect* 2015;**5**(2):104-15 doi: 10.15171/hpp.2015.013.

10 Drechsel P, Cofie OO, Van Veenhuizen R, et al. Linking research, capacity building, and policy dialogue in support of informal irrigation in urban West Africa. *Irrig Drain* 2008;**57**(3):268-78 doi: 10.1002/ird.430.

For peer review only

For peer review only - http://bmjopen.bmj.com/site/about/guidelines.xhtml

11 Babu SC, Ergeneman A. Policy Dialogue with Indian Parliamentarians: Outcomes and Lessons from the CGIAR. *J Int Commun* 2005;**11**(1):41-54

12 Nabyonga-Orem J, Dovlo D, Kwamie A, et al. Policy dialogue to improve health outcomes in low income countries: What are the issues and way forward? *BMC Health Serv Res* 2016;**16** doi: 10.1186/s12913-016-1450-2.

13 Kwamie A, Nabyonga-Orem J. Improved harmonisation from policy dialogue? Realist perspectives from Guinea and Chad. *BMC Health Serv Res* 2016;**16** doi: 10.1186/s12913-016-1458-7.

14 Molenaers N. The Great Divide? Donor perceptions of budget support, eligibility and policy dialogue. *Third World Q* 2012;**33**(5):791-806 doi: 10.1080/01436597.2012.677311.

15 Ade N, Réne A, Khalifa M, et al. Coordination of the health policy dialogue process in Guinea: Pre- and post-Ebola. BMC *Health Serv Res* 2016;**16** doi: 10.1186/s12913-016-1457-8.

16 White LG. Policy Reforms in Sub-Saharan Africa - Conditions for Establishing a Dialogue. *Stud Comp Int Dev* 1990;**25**(2):24-42

17 Scholten P, Verbeek S. Politicization and expertise: Changing research-policy dialogues on migrant integration in Europe. *Sci Public Policy* 2015;**42**(2):188-200 doi: 10.1093/scipol/scu040.

18 Lavis JN, Boyko JA, Gauvin FP. Evaluating deliberative dialogues focussed on healthy public policy. *BMC Public Health* 2014;**14**

19 Baumann C, White S. Collaborative Stakeholder Dialogue: A Catalyst for Better Transport Policy Choices. *Int J Sustain Transp* 2015;**9**(1):30-38

20 Susskind L, Hoben M. Making regional policy dialogues work: A credo for metro-scale consensus building. *Temp Envtl Law & Tech J* 2004:123-38

21 Perin E. Au coeur du dialogue social sectoriel européen : entre coordination européenne et négociations nationales : analyse de la mise en oeuvre des textes de nouvelle génération. Université catholique de Louvain, 2014.

For peer review only

For peer review only - http://bmjopen.bmj.com/site/about/guidelines.xhtml

22 Executive Board of the World Health Assembly. Strengthening national policy dialogue to build more robust health policies, strategies and plans. In: Executive Board of the World Health Assembly, ed. EB128.R12, 2011.

23 Pawson R, Tilley N. *Realistic evaluation*. London, United Kingdom: Sage Publications, 1997.

24 Lacouture A, Breton E, Guichard A, et al. The concept of mechanism from a realist approach: a scoping review to facilitate its operationalization in public health program evaluation. *Implement Sci* 2015;**10**(1):153 doi: 10.1186/s13012-015-0345-7.

25 Chen H-T, Rossi PH. The Multi-Goal, Theory-Driven Approach to Evaluation: A Model Linking Basic and Applied Social Science. *Soc Forces* 1980;**59**(1):106-22

26 Weiss C. How can theory-based evaluation make greater headway? *Eval Re* 1997;**21**(51):501-24 doi: 10.1177/0193841X9702100405.

27 Damani Z, MacKean G, Bohm E, et al. The use of a policy dialogue to facilitate evidence-informed policy development for improved access to care: The case of the Winnipeg Central Intake Service (WCIS). *Health Res Policy Syst* 2016;**14**(1) doi: 10.1186/s12961-016-0149-5.

28 Dhunpath R, Paterson A. The interface between research and policy dialogue: Substantive or symbolic? *Journal of Education* 2004;**33**:125-46.

29 Yin RK. *Case study research: Design and methods (Fifth edition)*. Thousand Oaks, CA: Sage, 2014.

30 Lasker RD, Weiss ES, Miller R. Partnership synergy: A practical framework for studying and strengthening the collaborative advantage. *Milbank Q* 2001;**79**(2):179-205.

31 Jones J, Barry MM. Exploring the relationship between synergy and partnership functioning factors in health promotion partnerships. *Health Promot Int* 2011;**26**(4):408-20 doi: 10.1093/heapro/dar002.

32 Jagosh J, Macaulay AC, Pluye P, et al. Uncovering the benefits of participatory research: Implications of a realist review for health research and practice. *Milbank Q* 2012;**90**(2):311-46 doi: 10.1111/j.1468-0009.2012.00665.x.

For peer review only

33 Christopoulos D, Ingold K. Exceptional or just well connected? Political entrepreneurs and brokers in policy making. *Eur Political Sci Rev* 2015;**7**(3):475-98 doi: 10.1017/S1755773914000277.

34 Pires A. Echantillonnage et recherche qualitative : essai théorique et méthodologique. In: Poupart J, Deslauriers J-P, Groulx L-H, Laperrière A, Mayer R, Pires AP, eds. La recherche qualitative: Enjeux épistémologiques et méthodologiques. Montréal, QC: Gaëtan Morin, 1997:113-69.

35 Varvasovszky Z, Brugha R. Stakeholder analysis: A review. *Health Policy Plan* 2000;**15**(3):239-46 doi: 10.1093/heapol/15.3.239.

36 Abimbola S, Molemodile SK, Okonkwo OA, Negin J, Jan S, Martiniuk AL, et al. 'The government cannot do it all alone': realist analysis of the minutes of community health committee meetings in Nigeria. *Health Policy Plan* 2016;**31**(3):332-45 doi: 10.1093/heapol/czv066.

37 Bhaskar R. *Reclaiming reality: A critical introduction to contemporary philosophy*. London: Verso, 1989.

38 Trimarchi M. Theory building: A realist methodology for case study driven research. Faculty of Business Working Paper Series. Sippy Downs, Australia: University of the Sunshine Coast, 1998:19-35.

39 Bhaskar R. *Scientific realism and human emancipation*. London, United Kingdom: Verso, 1986.

40 Wai-chung Yeung H. Critical realism and realist research in human geography: A method or a philosophy in search of a method? *Prog Hum Geogr* 1997;**21**(1):51-74.

41 McEvoy P, Richard D. A critical realist rationale for using a combination of quantitative and qualitative methods. *J Res Nurs* 2006;**11**(1):66-78 doi: 10.1177/1744987106060192.

42 Pawson R. *Evidence-based policy: A realist perspective*. London, United Kingdom: Sage Publications, 2006.

43 Nabyonga-Orem J, Ousman K, Estrelli Y, et al. Perspectives on health policy dialogue: Definition, perceived importance and coordination. *BMC Health Serv Res* 2016;**16** doi: 10.1186/s12913-016-1451-1.

For peer review only

LEGEND

Figure 1. Generic intervention theory of the UHC Partnership

For peer review only

For peer review only - http://bmjopen.bmj.com/site/about/guidelines.xhtml

AUTHORS' CONTRIBUTIONS

ER and VR designed the study. DR, OS, MD, and DP provided critical data and information regarding the intervention, the sampling of cases and feasibility of the research. ER drafted the first version of the protocol. All authors critically revised the study protocol for important intellectual content and approved the manuscript.

FUNDING STATEMENT

This work is funded by the Department of Health Systems Governance and Financing of the World Health Organization.

COMPETING INTERESTS STATEMENT

ER and VR are funded as research consultants by the Department of Health Systems Governance and Financing of the World Health Organization. DR, OS, MD and DP are experts at the World Health Organization, and involved within the Universal Health Coverage Partnership.

ACKNOWLEDGEMENTS

We would like to thank the following collaborators at WHO: Juliet Nabyonga, Martin Ota, and Kira Koch. We would also like to express our gratitude to the WHO country office and Sébastien Segniagbeto in Togo for their support during the pilot mission, as well as researchers in countries for support with national ethics committees. We finally would like to acknowledge Donna Riley for the translation.

For peer review only

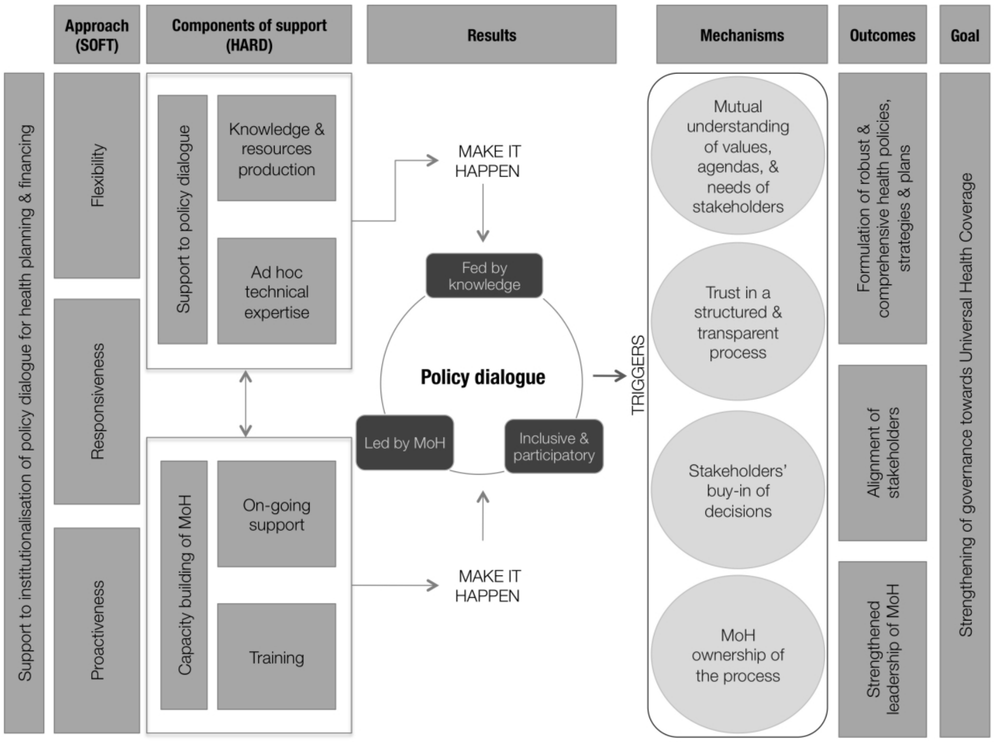

Figure 1 Intervention theory

1057x793mm (72 x 72 DPI)

Realist evaluation of the role of the Universal Health Coverage Partnership in strengthening policy dialogue for health planning and financing: A protocol

Journal:	*BMJ Open*
Manuscript ID	bmjopen-2018-022345.R1
Article Type:	Protocol
Date Submitted by the Author:	15-Aug-2018

Complete List of Authors:	Robert, Emilie; Research Institute of the McGill University Health Centre,<br>Ridde, Valery; Centre Population et Developpement; Universite de Montreal Institut de recherche en sante publique<br>Rajan, Dheepa; Organisation mondiale de la Sante, Department of Health Systems Governance and Financing<br>Sam, Omar; World Health Organization Inter-Country Support Team<br>Dravé, Mamadou; World Health Organization Country Office in Togo<br>Porignon, Denis; Organisation mondiale de la Sante, Department of Health Systems Governance and Financing

**Primary Subject Heading**:	Global health
Secondary Subject Heading:	Health policy, Qualitative research, Research methods
Keywords:	realist evaluation, policy dialogue, universal health coverage, health planning, health financing

SCHOLARONE™
Manuscripts

TITLE PAGE

Title of the article

Realist evaluation of the role of the Universal Health Coverage Partnership in strengthening policy dialogue for health planning and financing: A protocol

Full name, postal address, e-mail and telephone number of the corresponding author

Full name: Emilie Robert

Postal address: Institut de recherche du Centre universitaire de santé McGill

2155, rue Guy, 5e étage

Montréal (QC)

Canada H3H 2R9

E-mail: emilie.robert2@mail.mcgill.ca

Telephone number: +1.514.998.9892

Full name, department, institution, city and country of all co-authors

- Emilie Robert

  Research Institute of the McGill University Health Centre (RI-MUHC) (at the time of initial submission)
  Montreal, Canada

  AND

  Transcultural Research and Intervention Team
  SHERPA (Recherche. Immigration. Société)
  CSSS de la Montagne
  Montreal, Canada

- Valéry Ridde

  French Institute For Research on sustainable Development
  CEPED (IRD-Université Paris Descartes), Universités Paris Sorbonne Cités, ERL INSERM SAGESUD
  Paris, France

  AND

  University of Montreal Public Health Research Institute (IRSPUM)
  Montreal, Canada

For peer review only - http://bmjopen.bmj.com/site/about/guidelines.xhtml

- Dheepa Rajan

  Department of Health Systems Governance and Financing
  World Health Organization
  Geneva, Switzerland

- Omar Sam

  Inter-Country Support Team
  World Health Organization
  Ouagadougou, Burkina Faso

- Mamadou Dravé

  World Health Organization Office in Togo (at the time of the pilot mission)
  Lomé, Togo

- Denis Porignon

  Department of Health Systems Governance and Financing
  World Health Organization
  Geneva, Switzerland

Word count, excluding title page, abstract, references, figures and tables

4,057

For peer review only

ABSTRACT

Introduction

In 2011, the World Health Organization (WHO), the European Union and Luxembourg entered into a collaborative agreement to support policy dialogue for health planning and financing; these were acknowledged as core areas in need of targeted support in countries' quest towards universal health coverage (UHC). Entitled 'Universal Health Coverage Partnership', this intervention is intended to strengthen countries' capacity to develop, negotiate, implement, monitor, and evaluate robust and integrated national health policies oriented towards UHC. It is a complex intervention involving a multitude of actors working on a significant number of remarkably diverse activities in different countries.

Methods and analysis

The researchers will conduct a realist evaluation to answer the following question: How, in what contexts, and triggering what mechanisms, does the Partnership support policy dialogue for health planning and financing towards UHC? A qualitative multiple case study will be undertaken in Togo, Liberia, Democratic Republic of Congo, Cape Verde, Burkina Faso and Niger. Three steps will be implemented: 1) formulating Context-Mechanism-Outcome explanatory propositions to guide data collection, based on expert knowledge and theoretical literature; 2) collecting empirical data through semi-structured interviews with key informants and observations of key events, and analyzing data; 3) specifying the intervention theory.

Ethics and dissemination

The primary target audiences are WHO and its partner countries; international and national stakeholders involved in or supporting policy dialogues in the health sector, especially in low-income countries; and researchers with interest in UHC, policy dialogue, evaluation research, and/or realist evaluation. WHO-AFRO ethics review committee approved the study (AFR/ERC/2017/02.08). WHO as the funder of this research will not have any role in data collection and data analysis. However, any publication will require WHO approval.

KEY WORDS

Heath policy; realist evaluation; policy dialogue; universal health coverage; health planning; health financing

For peer review only

STRENGTHS AND LIMITATIONS OF THIS STUDY

1. The in-depth study of six countries ensures internal validity; and the potential to generalize the findings is increased by building explanations, taking the context into account in the production of outcomes, and using existing literature to provide theoretical foundations to the findings.
2. Methodological developments are expected, as the study is among the few realist evaluations to focus on policy dialogue, especially in low- and middle-income countries.
3. The study involves training and ongoing supervision of West African researchers who come from a different educational background, contributing to capacity building in health policy and systems research in low- and middle-income countries.
4. The study does not include countries or cases outside of the West African region.
5. Comprehensive data collection may be challenging in some countries with an unstable political situation.

For peer review only

For peer review only - http://bmjopen.bmj.com/site/about/guidelines.xhtml

INTRODUCTION

Universal health coverage (UHC) as a core objective within the Sustainable Development Goals is a journey in which multiple stakeholders, from the local, national and international levels, partake. It involves on-going discussions and negotiations among these stakeholders on the different facets of UHC, among which financing and planning questions.[1,2] Under the leadership of national health authorities, stakeholders will thus have to agree on priorities for action, and health financing and health system organization modalities.

Policy dialogue is where such discussions take place. The World Health Organization (WHO) describes policy dialogue as an iterative process that targets both the technical and policy aspects of the problem being discussed, involving evidence and sensitive policy discussions, in which a wide range of stakeholders participate.[3] This dialogue has a concrete objective, such as the development of a plan, a strategy or a policy. Policy dialogue is thus understood as a deliberative process by which different stakeholders are brought together to discuss issues of public policy to feed into decision-making.

In low- and middle-income country (LMIC) settings, the topic area of policy dialogue is attracting interest as a target intervention for programmes. Policy dialogue is seen as a key governance tool to ensure the coordination and alignment of stakeholders and to develop robust and comprehensive health policies anchored in the UHC concept.[4] Negotiations in the health sector may indeed be challenging due to the heavy influence external aid can have on national priorities, combined with the often limited government steering capacity to counter-balance that influence.[5,6] In the context of UHC, Ministries of Health's convening and brokering role takes on an added layer of importance, as they need to bring together a diverse range of interests to mobilize resources, prioritize interventions, and strengthen institutions for UHC.

Following this trend, policy dialogue is becoming a popular research object.[7] In the field of health policy and systems research in LMICs, scholars study policy dialogue as a knowledge translation instrument,[8-11] or as a decision-making process. In this case, they notably study the perceptions of what policy dialogue is.[12-14] The actual process of policy dialogue is more rarely investigated.[15,16] In high-income countries, research on policy dialogue as part of decision-making is more diffuse and includes research on research-policy dialogue,[17,18] stakeholder dialogue,[19,20] or social dialogue.[21]

In 2011, WHO, the European Union and Luxembourg entered into a collaborative agreement called the *Universal Health Coverage Partnership* to support policy dialogue for health planning and financing as core topic areas of UHC. This Partnership also responds to a resolution from the 64th World Health Assembly calling on WHO to support countries in developing more robust and evidence-based health policies and plans based on an inclusive and participatory process.[22] Implemented in 28 countries, the Partnership is a complex intervention involving a multitude of actors working on a significant number of very diverse activities in different countries. Moreover, it takes place in the policy arena where policy-making is complex and dynamic. This study aims to examine the Partnership through a realist lens, to better understand how and in what contexts it can, through WHO in-country support, contribute to strengthening policy dialogue.

For peer review only - http://bmjopen.bmj.com/site/about/guidelines.xhtml

REALIST APPROACH

Traditional evaluation methods face three limitations when studying complex interventions in dynamic contexts, as is the case of the Partnership. First, establishing a causal relationship between the Partnership and its expected outcomes in terms of policy formulation and donors alignment on one hand, and between the Partnership and the indicators usually used in classical evaluations on the other, is onerous as such relationships are not direct and are strongly colored by the contexts within which WHO intervenes. Second, WHO's role in the Partnership complements its traditional role as technical advisor. Demonstrating how this new approach helps support policy dialogue, and painting a clear picture of the challenges involved, is difficult to do using traditional evaluation methods. Finally, the Partnership has been implemented in several countries representing a wide variety of contexts, which raises the third limitation of traditional evaluations: the inability to take context into account in explaining an intervention's outcomes and to draw parallels between the experiences of different countries. Therefore, the realist approach was deemed most appropriate as a frame to evaluate the UHC Partnership.

A realist evaluation entails the premise that an intervention does not function on its own and is not, in itself, what produces an outcome.[23] Instead, an outcome is the product of the interaction between a mechanism triggered by the intervention and the context in which the intervention is implemented. A mechanism should be understood as the way in which the actors involved in an intervention react to and reason about the resources made available to them as part of the intervention.[24]

A realist evaluation, therefore, does not attempt to control the variables associated with the implementation context. Rather, it seeks to observe, in the natural environment, the regular—although not systematic—occurrence of an outcome in a particular context, when a mechanism is triggered. These iterations of interactions are referred to as context–mechanism–outcome (CMO) configurations. The objective of a realist evaluation is thus to identify, in the empirical data, iterative interactions between an intervention's context, mechanisms, and outcomes. Operationally, it begins with a reflection on the intervention theory and results in the production of an explanatory theory, which is sufficiently abstract to explain how the intervention functions in different contexts. This anchoring in theory requires an explicitly iterative process between the conceptual and theoretical literature underlying the rationale behind the intervention, and the empirical data.

INTERVENTION THEORY

Several sources of information were used to develop an initial intervention theory of the Partnership. In addition to reviewing the documents produced by WHO, several meetings were held with stakeholders from WHO headquarters' Department of Health Systems Governance and Financing which implements the Partnership. These meetings aimed to make explicit the logical reasoning underlying the need for and the rationale behind the Partnership. Observations of two annual inter-country meetings bringing together partner countries and WHO teams were also conducted. This process resulted in a generic intervention theory that was discussed at a workshop in Burkina Faso in 2017 and subsequently adjusted to take into account the participants' input (Figure 1).

For peer review only - http://bmjopen.bmj.com/site/about/guidelines.xhtml

In the frame of the Partnership, WHO supports policy dialogue processes for health planning and health financing. According to WHO, such processes should be inclusive and participatory, fed by knowledge and evidence, and led by the Ministries of Health (MoH). WHO's role is to support MoH to lead structured and transparent policy dialogues. The ultimate goal is to contribute to strengthening health systems, especially its governance function, with the assumption that health systems strengthening is the principal means to achieve UHC. WHO support through the Partnership manifests itself through a hard component, including financial resources for a set of activities as well as human resources, and a soft component, which refers to a specific approach to supporting the policy dialogue. The hard component of WHO support includes strengthening of the capacities of MoH to conduct policy dialogue processes. This involves WHO country office on-going support as well as training. It also involves strengthening of the quality of the policy dialogue itself with the generation of relevant evidence, data and normative guidelines, as well as *ad hoc* expertise. With regard to WHO's approach, three characteristics are essential: flexibility, i.e. the ability to adapt to the changing priorities of the MoH; responsiveness, or the ability to position and respond to requests from the MoH; and proactivity, i.e. the ability to anticipate implementation steps and expectations or needs of the MoH.

The resulting policy dialogue is expected to enable the formulation of robust and comprehensive health policies, strategies and plans that promote the principles of UHC such as equity, financial risk protection, and quality of care. It should also facilitate the alignment of multiple stakeholders, be they donors, civil society, or related ministries. Thus, the Partnership is supposed to contribute to upholding the principles of the Paris Declaration to ensure effective development cooperation. Finally, supporting MoH in the organization of such dialogues is expected to strengthen the capacities and leadership position of the MoH. To summarize, WHO has three principal roles within the Partnership: convener of policy dialogue processes, policy broker, and a relatively traditional role of advisor to the MoH.

(Insert Figure 1)

WHO may act as a "boundary spanner", i.e., an actor who brings together partners with common interests and goals (such as UHC), while striving to establish a climate of trust, optimism, and perseverance.[25] The concept of policy broker is similar to that of boundary spanner. The role of a broker is to bring together groups of actors whose interests and beliefs differ about the issue of concern in the policy dialogue.[26] The broker's objective is to promote consensus in a climate of trust, to stabilize the policy dialogue and to find workable compromises. However, his role is not necessarily a disinterested one, and he might be pursuing a strategic objective, which would bring him closer to the role of a policy entrepreneur. A policy entrepreneur has an explicit strategic objective, and proposes ideas and stimulates opportunities in ways that are intended to create a demand for the solution he is proposing.[26] These roles are, however, ideal types positioned at two ends of a continuum.[26] Depending upon the contexts in which it intervenes, the processes in which it is involved, and the issues being discussed, WHO may be called upon to play one or another of these various roles.

For peer review only - http://bmjopen.bmj.com/site/about/guidelines.xhtml

METHODS AND ANALYSIS

RESEARCH QUESTIONS

The question that will guide the study is: How, in what contexts, and triggering what mechanisms does the Partnership support policy dialogue for health planning and health financing towards UHC, and with what outcomes?

Four specific objectives have been established to respond to this research question:

1) To highlight the contexts in which the Partnership, through WHO support, can, or cannot:

- act as a broker, promoting the dissemination and use of data and evidence in policy dialogue and seeking workable compromises;
- act as a convener, creating synergy among the actors involved in policy dialogue for health planning and financing;
- play its role as an advisor, ensuring transparent and structured policy dialogue in accordance with available evidence and equity principles; and supporting MoH in its leadership and stewardship functions.

2) To identify the mechanisms at work and the outcomes—direct or indirect, expected or not—produced;

3) To clarify the process chains and the links between them, especially highlighting feedback loops;

4) To specify how, in what contexts, triggering what mechanisms, and with what outcomes the Partnership, through WHO support, enhances policy dialogue for health planning and financing towards UHC.

STUDY DESIGN

A realist multiple case study will be undertaken.[27] Table 1 presents a summary of the proposed design. The countries in which the study will be conducted are Togo, Liberia, Democratic Republic of Congo, Cape Verde, Burkina Faso and Niger. Due to feasibility concerns, only partner countries of the African continent were selected. They were sampled using a non-probabilistic approach. The objective was to select cases—or countries—purposively, so as to obtain a diversity and depth of information on the Partnership. This is therefore a contrasted sample. When selecting cases, purposive sampling involves establishing selection criteria that ensure contrasting cases. The criteria are based on theoretical assumptions about the influence they may have on the Partnership and policy dialogue. An initial list was shared with WHO in order to select the criteria that could be informed by their experience. Based on their country knowledge and experience, they then suggested countries matching the criteria, based on their country knowledge and experience (Table 2).

For peer review only - http://bmjopen.bmj.com/site/about/guidelines.xhtml

Table 1. Summary of the study design

**Epistemological foundation**	Critical realism
**Design**	Multiple case studies
**Cases**	Countries involved in the Partnership
**Units of analysis**	Policy dialogue processes supported by WHO as part of the country roadmaps
**Type of sample**	Contrasted
**Case sampling strategy**	Purposive sampling

Each country has a roadmap for UHC Partnership work, in line with its needs. In this study, the unit of analysis is a policy dialogue process defined in the country's roadmap. One process (unit of analysis) will be observed in each country (case). Each process was selected in collaboration with the country teams, taking into account on-going support at the start of data collection, as well as opportunities for observation. In Togo and DRC, the processes under study are the policy dialogues around the national health financing strategy, whereas in Cape Verde, Niger and Togo, there are the policy dialogues for the elaboration of the national or regional health plans. In Liberia, the process under study is the elaboration of the IHP+ (International Health Partnership) Compact, which is an aid coordination agreement amongst health sector stakeholders. The process selected determines the research sub-questions to be addressed in each case, the documentation to be obtained, the informants to interview, and the topics to be covered in interviews.

For peer review only - http://bmjopen.bmj.com/site/about/guidelines.xhtml

Table 2. Case selection criteria and reason for sampling

Criteria*	Togo	Liberia	DRC	Cape Verde	Burkina Faso	Niger
Duration of WHO support	4	4	3	3	3	3
Political stability (0=instable; 3=stable)	3	3	2	3	3	1,5
Ministerial stability (0=instable; 3=stable)	3	3	3	2	2	1
Openness to policy dialogue (0=not open; 3=open)	3	3	3	2	2	1,5
Ministry of Health leadership capacity (0=weak; 3=strong)	3	3	3	3	3	1,5
Implementation of the national road map (0=weak; 3=good)	3	3	3	2	3	1
Presence of entry points	Yes	Yes	Yes	Yes	Yes	Yes
Additional observations		*Ebola epidemic*	*Devolution*	*Archipelago – Lower middle-income country*		

** At the time of case selection*

Note: Criteria were noted on a scale from 1 to 3, where 1 is the lowest and 3 the highest. For example, a score of 3 for Togo for criteria 'Duration of WHO support' means that the country benefited from WHO support for the longest period of time.

STUDY STEPS

Step 1: Formulating context-mechanism-outcome propositions

Within a realist perspective, researchers raise the questions of the mechanisms, that is, how actors react to the new resources made available through an intervention, and of the contexts that trigger or hinder these mechanisms. The initial intervention theory was divided into two sub-theories to clarify what mechanisms and contexts may interact (Figure 2 and Figure 3). Contexts and mechanisms derive from three sources: literature on partnership synergy;[25,28,29] a scoping study on policy dialogue,[30] looking into knowledge translation, policy change, and development studies literature; and interviews conducted with international experts involved in the Partnership. We describe below both sub-theories, highlighting mechanisms (M), outcomes (O), and potential contexts (C).

The first sub-theory focuses on the Partnership and its expected outcomes (Figure 2). It posits that capacity building, through training, technical expertise and on-going support from WHO, would empower MoH (M), while triggering a shared understanding of governance and policy dialogue (M). This should result in MoH leading inclusive and participatory policy dialogues (O). Furthermore, the on-going support provided to MoH, combined with WHO approach (i.e. proactiveness, responsiveness, flexibility), should prompt mutual trust (M), which in turn should strengthen their collaboration for policy dialogues (O). Finally, the sub-theory postulates that the

For peer review only - http://bmjopen.bmj.com/site/about/guidelines.xhtml

availability of evidence and data provided through the Partnership would bring about a shared understanding of the needs and policy options by MoH and WHO (M), contributing to an evidence-informed policy dialogue (O). In sub-theory 1, mechanisms may be triggered by the Partnership when: existing national governance arrangements facilitate the organization of policy dialogue (C); WHO and MoH have enduring relationships (C); they have the expected expertise and skills (C); they both endorse collaborative and cross-sectoral ways of working (C); human resources from both institutions who are involved in the Partnership are stable (C); and there is synergy between the Partnership and international stakeholders' priorities and plans to strengthen MoH leadership (C).

(Insert Figure 2)

The second sub-theory focuses on policy dialogue and its expected outcomes (Figure 3). It posits that an evidence-informed policy dialogue would first trigger a shared understanding among stakeholders of the challenges and possible ways of action (M). This should contribute to more robust and comprehensive health policies, strategies and plans (O) through the formulation of commonly agreed workable propositions. An inclusive and participatory policy dialogue should also prompt a shared understanding of stakes, values and interests of the various actors involved (M), as well as buy-in of the decisions (M), strengthening stakeholders' alignment on national health priorities (O). Finally, brought under the responsibility of MoH, it should foster MoH sense of ownership of the process (M), contributing to strengthening its leadership (O). Such an outcome may also be the consequence of stakeholders' trust in a structured and transparent policy dialogue process (M). In sub-theory 2, policy dialogue may trigger such mechanisms when: participants show openness to policy dialogue (C); they have the capacity and legitimacy to actively participate (C); they have previous positive experience of policy dialogue (C); their organizations' culture value collaboration and cross-sectoral ways of working (C); policy dialogue benefit from financial and technical support (e.g. the Partnership) (C); there is synergy between the global and national health agendas (C); and policy dialogue matches socio-cultural forms of communication and participation to governance and formal events (C).

(Insert Figure 3)

For each case, we will formulate at least one CMO proposition for each outcome, based on these theories. Additional theoretical literature may be used to fill in potential knowledge gaps, such as capacity building literature and theories about organizational change.

Step 2: Collecting and analyzing empirical data

Once the propositions are developed, an interview guide will be drafted for each case. The interviewee is there to help the researcher 'refine' the theory, as each interviewee holds a piece of the complex puzzle of the intervention.[23] Consequently, the interview guide will be adjusted to the interviewee. For example, it is expected that WHO informants will be knowledgeable about the Partnership and its outcomes (sub-theory 1), while other stakeholders will be able to share their experience as policy dialogue participants (sub-theory 2).

Three sources of information will be used. First, semi-structured interviews will be conducted with key informants who will be jointly identified by the researchers and WHO. An initial list

For peer review only - http://bmjopen.bmj.com/site/about/guidelines.xhtml

will be compiled by WHO and then supplemented using a snowballing technique. The aim is to meet with as many as possible of the participants of the policy dialogue and avoid investigating merely the positive side of the coin, and thus possible bias. Depending on informants' availability, interviews will be conducted in person or by telephone. The interviews will be audio-recorded, except when not allowed by the participants. They will also be recorded in notes taken systematically by the researchers. Recordings will subsequently be transcribed. Between 20 and 30 informants will be interviewed for each case. The principle of empirical saturation will be applied to determine the best time to bring the data collection to a close.[31]

Observations will also be conducted of activities organized either directly by WHO or with WHO collaboration within the Partnership context. These observations will be described and recorded in the logbooks of researchers. Also, all documentation related to the Partnership (e.g. national roadmaps, project documents, annual reports) will be reviewed.

Furthermore, to gain a deeper understanding of the national health policy contexts, and particularly of the interests, power relationships, and positioning of the actors involved in policy dialogue, a stakeholder analysis will be performed for each case.[32] First, we will compile a list of stakeholders involved in policy dialogue for each case. Then, for each stakeholder, we will attempt to describe their power of action and influence, as well as their interest and involvement with respect to UHC and policy dialogue. The last step will be to analyze how these stakeholders react to the roles played by WHO in policy dialogue, and how their reactions influence these two processes. This exercise will be conducted before the interviews in collaboration with the WHO teams, who have first-hand knowledge of the field. The analysis will be completed during the interviews with the stakeholders included on the list.

Using an iterative approach during data collection, we will deepen our understanding of the intervention theory, including "hypotheses about their subjects' reasoning within a wider model of their causes and consequences" (p.163).[23] Data analysis will contribute to deepening our understanding further. It will start with a thorough description of the barriers and facilitators for each piece of the intervention theory. We will then move to identify in the data the interaction between context, mechanisms, and outcomes. Finally, we will attempt to identify - across interviews - patterns of such interactions, called context-mechanism-outcome (CMO) configurations. Both barriers and facilitators and CMO configurations will be identified in the data through content analysis performed using NVivo software. For the latter, we will: 1) identify the outcomes (description); 2) determine the contextual components related to the outcomes (resolution); 3) reconstruct the links between context and outcomes theoretically (abduction); 4) identify the mechanisms (retroduction).[33] Retroduction aims to construct the theoretical explanation of a phenomenon.[34,35] More specifically, this retrospective reasoning allows moving from describing the outcome to describing what produced that outcome and the context within which it takes place,[36,37] via the mechanisms.[38] In case of missing pieces of evidence, additional interviews will be conducted whenever possible.

Step 3: Specifying the theory

In order to specify the intervention theory across cases, a transversal analysis will be conducted using C-M-O configurations as primary data. Such configurations may be incomplete, with the mechanism generally not being explicit. The missing links will thus be inferred from the

For peer review only - http://bmjopen.bmj.com/site/about/guidelines.xhtml

theoretical literature, which provides clues to explain the links between causes and outcomes, following a process of intellectual reasoning based on two questions: How can we explain the mechanism in this context and considering this outcome? Is this interpretation supported by the theoretical literature?

This inference takes the form of a demi-regularity when the configuration re-appears several times. A demi-regularity is a regular—although not systematic—occurrence in the interactions among the context, the mechanism(s), and the outcomes.[39] When a configuration is identified only once but its interpretation is supported by the theoretical literature, it is also taken into account, on the condition that it helps to clarify how and under what circumstances the Partnership contributes to each outcome. Each demi-regularity will provide one or more piece of information that, when combined, present a more complete and precise picture of the Partnership. Such an analysis will contribute to specify the explanatory realist theory of the Partnership. Table 3 summarizes the study process, including sources of data, methods and status. The research should be completed by mid-2019.

Table 3. Summary of the study process

Steps	Data sources	Methods	Status
Building a generic intervention theory	▪ Program documents<br>▪ Meetings with stakeholders from WHO headquarters' Department of Health Systems Governance and Financing<br>▪ Observations of annual inter-country meetings (n=2)	Iterative co-building process	Completed (2016)
Formulating CMO explanatory propositions	▪ Literature reviews on policy dialogue<br>▪ Semi-structured interviews with experts at WHO headquarters and at regional level (n=13)	▪ Scoping study<br>▪ Stakeholders analysis<br>▪ Content analysis	On-going (2017-18)
Identifying CMO configurations	▪ Semi-structured interviews with WHO national experts, MoH counterparts, participants of policy dialogues (e.g. civil society, international stakeholders, connected Ministries and public institutions)<br>▪ Observations of policy dialogues	Content analysis	2018
Specifying intervention theory	▪ CMO configurations from cases	Transversal analysis	2019

PATIENTS AND PUBLIC INVOLVEMENT

There is no patient or public involvement in this study.

ETHICS AND DISSEMINATION

PROTECTION OF KEY INFORMANTS

For peer review only - http://bmjopen.bmj.com/site/about/guidelines.xhtml

The research objectives and a consent form will be sent by email to informants before the interviews. The rights of informants include the right to refuse to participate in the interview, to end their participation in the study at any time, and to not allow their statements to be audio-recorded. Informed consent will be sought in writing from informants. We expect that all informants will be literate, given that they occupy positions of responsibility.

Informants' statements will be kept confidential. The data collected will be kept anonymous at the time of transcription, i.e., informants' names will be concealed, as well as their title and their function. Transcriptionists and everyone involved in data collection and analysis will sign confidentiality agreements. A copy of the data will be maintained on a password-protected virtual platform. The data will be destroyed 18 months after the signing of the consent forms.

ETHICS APPROVAL OF RESEARCH

The research protocol has been approved by the Ethics Committee of the WHO Regional office for Africa (ARF/ERC/2017/02.08), as well as the Ethics Committee of each country included in the study.

IMPORTANCE OF THE STUDY

First, in providing lessons on how the Partnership works and what proximal and unexpected outcomes it produces, the research should allow WHO and Partnership donors to improve the Partnership content, to adapt it better to the contexts in which it is implemented, and to tailor the nature of the policy dialogue support to take into account the diverse influences that foster or undermine productive collaboration among stakeholders. This more constructive collaboration should in turn contribute to more robust and actionable health policies, as a means to strengthen health systems so that they can deliver the quality and affordable health services users are entitled to under the banner of UHC.

Second, the realist evaluation has been little used to study interventions on an international scale. Thus, this study will be a unique example in health policy and systems research, and will offer important methodological lessons. Furthermore, it entails the training and ongoing supervision of a team of West African researchers coming from various educational backgrounds and with different expertise. This project will help strengthen capacities in health policy and systems research in low- and middle-income countries.

Finally, in terms of the topics addressed, the nature of the knowledge produced will be unique and will provide a deeper understanding of the issues associated with interventions aimed to support policy dialogue. As such, it adds to the findings on policy dialogue reported in the *BMC Health Services Research* special issue.[40]

DISSEMINATION OF RESULTS

The results of the study will be disseminated on several levels: WHO, including the headquarters, the regional teams, and the country teams; WHO's national and international partners, particularly MoH and donors; and the scientific community. With regards to the scientific community, empirical findings and methodological issues will be shared in related

For peer review only

symposia and in peer-reviewed journals. Any intention to publish the findings must be approved by WHO before submitting to the journal.

For peer review only

For peer review only - http://bmjopen.bmj.com/site/about/guidelines.xhtml

REFERENCES

1. Schmets G, Rajan D, Kadandale S. Strategizing national health in the 21st century: a handbook. Geneva: World Health Organization, 2016.

2. Evans T, Pablos-Mendez A. Shaping of a new era for health financing. *Lancet* 2016;**387**(10037):2482-4. doi: 10.1016/S0140-6736(16)30238-0

3. World Health Organization. *Health Systems Strengthening: Glossary*. Geneva, Switzerland: World Health Organization, 2011.

4. Rajan D, Adam T, El Husseiny D, et al. *Briefing Note - Policy dialogue: What it is and how it can contribute to evidence-informed decision-making*. Geneva: WHO, 2015.

5. Uneke CJ, Ezeoha AE, Ndukwe CD, et al. Enhancing leadership and governance competencies to strengthen health systems in Nigeria: assessment of organizational human resources development. *Healthc Policy* 2012;**7**(3):73-84.

6. Quinn M. *Governance and Health in Post-Conflict Countries: The Ebola Outbreak in Liberia and Sierra Leone*. New York: International Peace Institute, 2016.

7. Nabyonga-Orem J, Ota M, Mwisongo A, et al. Health policy dialogue: lessons from Africa. *BMC Health Serv Res* 2016;**16**(Supplement 4)

8 Lavis JN, Boyko J, Oxman AD, et al. SUPPORT Tools for evidence-informed health Policymaking (STP) 14: Organising and using policy dialogues to support evidence-informed policymaking. *Health Res Policy Syst* 2010;**10**(5):514-19 doi: 10.1186/1478-4505-7-S1-S14.

9. Uneke CJ, Ebeh Ezeoha A, Uro-Chukwu H, et al. Promoting Evidence to Policy Link on the Control of Infectious Diseases of Poverty in Nigeria: Outcome of A Multi-Stakeholders Policy Dialogue. *Health Promot Perspect* 2015;**5**(2):104-15. doi: 10.15171/hpp.2015.013

10. Drechsel P, Cofie OO, Van Veenhuizen R, et al. Linking research, capacity building, and policy dialogue in support of informal irrigation in urban West Africa. *Irrig Drain* 2008;**57**(3):268-78. doi: 10.1002/ird.430

11. Babu SC, Ergeneman A. Policy Dialogue with Indian Parliamentarians: Outcomes and Lessons from the CGIAR. *J Int Commun* 2005;**11**(1):41-54.

12. Nabyonga-Orem J, Dovlo D, Kwamie A, et al. Policy dialogue to improve health outcomes in low income countries: What are the issues and way forward? *BMC Health Serv Res* 2016;**16** doi: 10.1186/s12913-016-1450-2

13. Kwamie A, Nabyonga-Orem J. Improved harmonisation from policy dialogue? Realist perspectives from Guinea and Chad. *BMC Health Serv Res* 2016;**16** doi: 10.1186/s12913-016-1458-7

For peer review only - http://bmjopen.bmj.com/site/about/guidelines.xhtml

14. Molenaers N. The Great Divide? Donor perceptions of budget support, eligibility and policy dialogue. *Third World Q* 2012;**33**(5):791-806. doi: 10.1080/01436597.2012.677311

15. Ade N, Réne A, Khalifa M, et al. Coordination of the health policy dialogue process in Guinea: Pre- and post-Ebola. *BMC Health Serv Res* 2016;**16** doi: 10.1186/s12913-016-1457-8

16. White LG. Policy Reforms in Sub-Saharan Africa - Conditions for Establishing a Dialogue. *Stud Comp Int Dev* 1990;**25**(2):24-42.

17. Scholten P, Verbeek S. Politicization and expertise: Changing research-policy dialogues on migrant integration in Europe. *Sci Public Policy* 2015;**42**(2):188-200. doi: 10.1093/scipol/scu040

18. Lavis JN, Boyko JA, Gauvin FP. Evaluating deliberative dialogues focussed on healthy public policy. *BMC Public Health* 2014;**14**

19. Baumann C, White S. Collaborative Stakeholder Dialogue: A Catalyst for Better Transport Policy Choices. *Int J Sustain Transp* 2015;**9**(1):30-38.

20. Susskind L, Hoben M. Making regional policy dialogues work: A credo for metro-scale consensus building. *Temp Envtl Law & Tech J* 2004:123-38.

21. Perin E. Au coeur du dialogue social sectoriel européen : entre coordination européenne et négociations nationales : analyse de la mise en oeuvre des textes de nouvelle génération. Université catholique de Louvain, 2014.

22. Executive Board of the World Health Assembly. *Strengthening national policy dialogue to build more robust health policies, strategies and plans*. In: Executive Board of the World Health Assembly, ed. EB128R12, 2011.

23. Pawson R, Tilley N. Realistic evaluation. London, United Kingdom: Sage Publications, 1997.

24. Lacouture A, Breton E, Guichard A, et al. The concept of mechanism from a realist approach: a scoping review to facilitate its operationalization in public health program evaluation. *Implement Sci* 2015;**10**(1):153. doi: 10.1186/s13012-015-0345-7

25. Jones J, Barry MM. Exploring the relationship between synergy and partnership functioning factors in health promotion partnerships. *Health Promot Int* 2011;**26**(4):408-20. doi: 10.1093/heapro/dar002

26. Christopoulos D, Ingold K. Exceptional or just well connected? Political entrepreneurs and brokers in policy making. *Eur Political Sci Rev* 2015;**7**(3):475-98. doi: 10.1017/S1755773914000277

27. Yin RK. Case study research: Design and methods (Fifth edition). Thousand Oaks, CA: Sage, 2014.

For peer review only

28. Lasker RD, Weiss ES, Miller R. Partnership synergy: a practical framework for studying and strengthening the collaborative advantage. *Milbank Q* 2001;**79**(2):179-205, III-IV.

29. Jagosh J, Macaulay AC, Pluye P, et al. Uncovering the Benefits of Participatory Research: Implications of a Realist Review for Health Research and Practice. *Milbank Q* 2012;**90**(2):311-46.

30. Robert E, Ridde V. « Policy dialogue » (dialogue sur les politiques) : une synthèse exploratoire des écrits scientifiques. *Cahiers REALISME* 2018;**(sous presse)**(16)

31. Pires A. Echantillonnage et recherche qualitative : essai théorique et méthodologique. In: Poupart J, Deslauriers J-P, Groulx L-H, et al., eds. La recherche qualitative: Enjeux épistémologiques et méthodologiques. Montréal, QC: Gaëtan Morin 1997:113-69.

32. Varvasovszky Z, Brugha R. Stakeholder analysis: A review. *Health Policy Plan* 2000;**15**(3):239-46. doi: 10.1093/heapol/15.3.239

33. Abimbola S, Molemodile SK, Okonkwo OA, et al. 'The government cannot do it all alone': realist analysis of the minutes of community health committee meetings in Nigeria. *Health Policy Plan* 2016;**31**(3):332-45. doi: 10.1093/heapol/czv066

34. Bhaskar R. Reclaiming reality: A critical introduction to contemporary philosophy. London: Verso, 1989.

35. Trimarchi M. *Theory building: A realist methodology for case study driven research*. Faculty of Business Working Paper Series. Sippy Downs, Australia: University of the Sunshine Coast, 1998:19-35.

36. Bhaskar R. Scientific realism and human emancipation. London, United Kingdom: Verso, 1986.

37. Wai-chung Yeung H. Critical realism and realist research in human geography: A method or a philosophy in search of a method? *Prog Hum Geogr* 1997;**21**(1):51-74.

38. McEvoy P, Richard D. A critical realist rationale for using a combination of quantitative and qualitative methods. *J Res Nurs* 2006;**11**(1):66-78. doi: 10.1177/1744987106060192

39. Pawson R. Evidence-based policy: A realist perspective. London, United Kingdom: Sage Publications, 2006.

40. Nabyonga-Orem J, Ousman K, Estrelli Y, et al. Perspectives on health policy dialogue: Definition, perceived importance and coordination. *BMC Health Serv Res* 2016;**16** doi: 10.1186/s12913-016-1451-1

For peer review only - http://bmjopen.bmj.com/site/about/guidelines.xhtml

LEGEND

Figure 1. Generic intervention theory of the UHC Partnership

Figure 2. Initial sub-theory of UHC Partnership (sub-theory 1)

Figure 3. Initial sub-theory of policy dialogue (sub-theory 2)

For peer review only

For peer review only - http://bmjopen.bmj.com/site/about/guidelines.xhtml

AUTHORS' CONTRIBUTIONS

ER and VR designed the study. DR, OS, MD, and DP provided critical data and information regarding the intervention, the sampling of cases and feasibility of the research. ER drafted the first version of the protocol. All authors critically revised the study protocol for important intellectual content and approved the manuscript.

FUNDING STATEMENT

This work is funded by the Department of Health Systems Governance and Financing of the World Health Organization.

COMPETING INTERESTS STATEMENT

ER and VR are funded as research consultants by the Department of Health Systems Governance and Financing of the World Health Organization. DR, OS, MD and DP are experts at the World Health Organization, and involved within the Universal Health Coverage Partnership.

ACKNOWLEDGEMENTS

We would like to thank the following collaborators at WHO: Juliet Nabyonga, Martin Ota, and Kira Koch. We would also like to express our gratitude to the WHO country office and Sébastien Segniagbeto in Togo for their support during the pilot mission, as well as researchers in countries for support with national ethics committees. We finally would like to acknowledge Donna Riley for the translation.

For peer review only

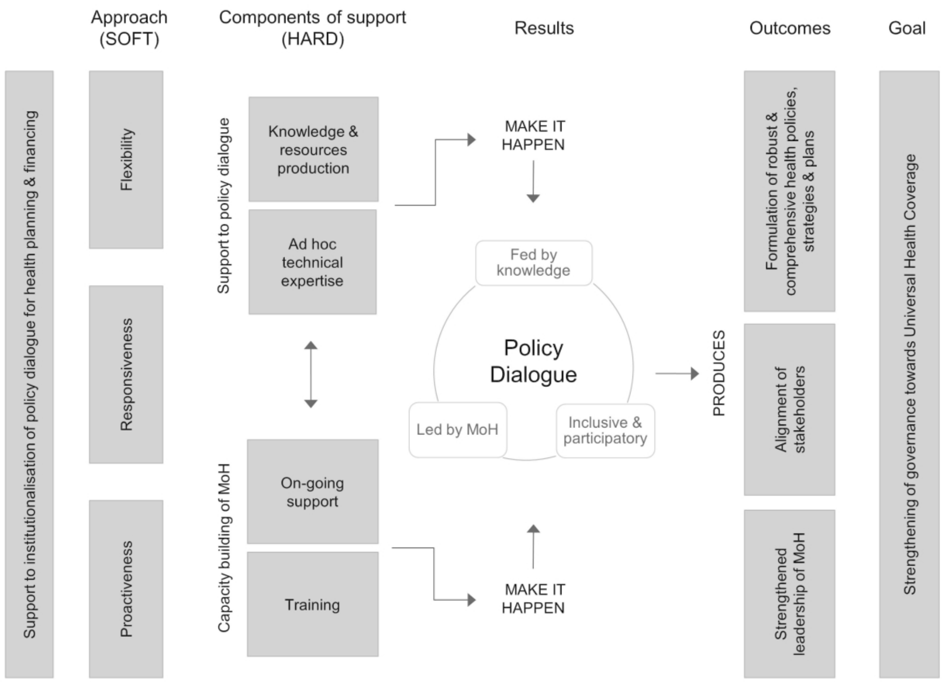

Caption : Generic intervention theory of the UHC Partnership

254x190mm (300 x 300 DPI)

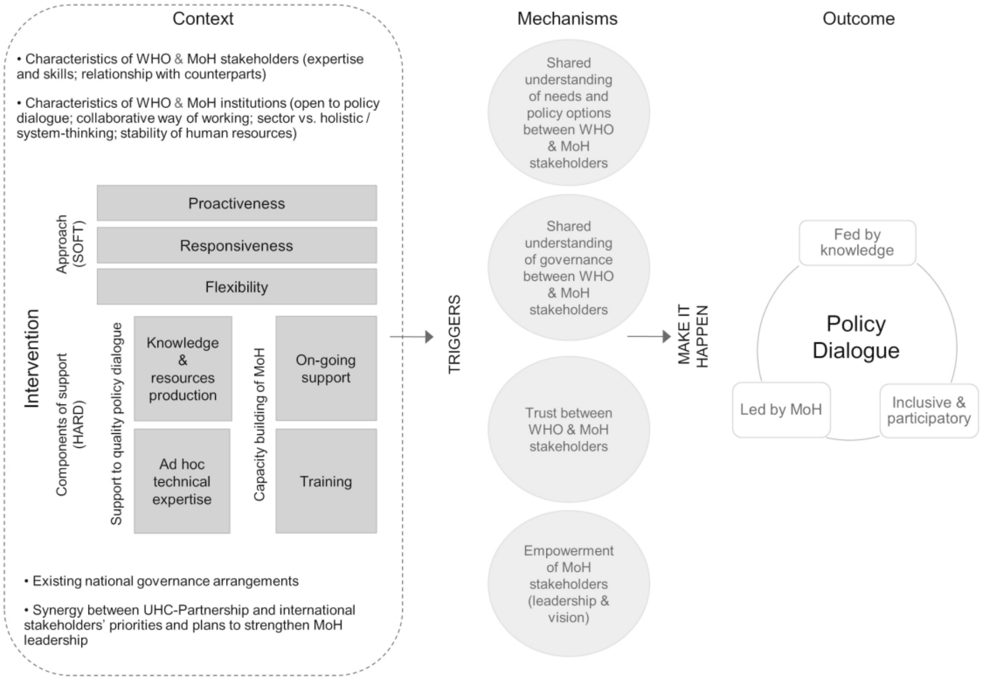

Caption : Initial sub-theory of UHC Partnership (sub-theory 1)

254x190mm (300 x 300 DPI)

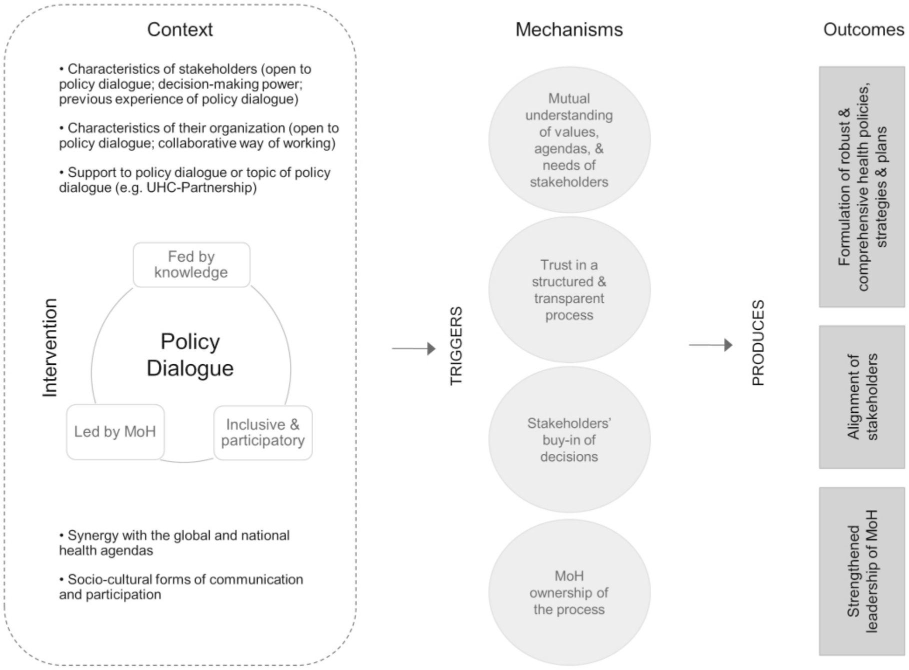

Caption : Initial sub-theory of policy dialogue (sub-theory 2)

254x190mm (300 x 300 DPI)

BMJ Open

Realist evaluation of the role of the Universal Health Coverage Partnership in strengthening policy dialogue for health planning and financing: A protocol

Journal:	*BMJ Open*
Manuscript ID	bmjopen-2018-022345.R2
Article Type:	Protocol
Date Submitted by the Author:	08-Oct-2018
Complete List of Authors:	Robert, Emilie; Research Institute of the McGill University Health Centre,<br>Ridde, Valery; Centre Population et Developpement; Universite de Montreal Institut de recherche en sante publique<br>Rajan, Dheepa; Organisation mondiale de la Sante, Department of Health Systems Governance and Financing<br>Sam, Omar; World Health Organization Inter-Country Support Team<br>Dravé, Mamadou; World Health Organization Country Office in Togo<br>Porignon, Denis; Organisation mondiale de la Sante, Department of Health Systems Governance and Financing
**Primary Subject Heading**:	Global health
Secondary Subject Heading:	Health policy, Qualitative research, Research methods
Keywords:	realist evaluation, policy dialogue, universal health coverage, health planning, health financing

SCHOLARONE™
Manuscripts

For peer review only - http://bmjopen.bmj.com/site/about/guidelines.xhtml

TITLE PAGE

Title of the article

Realist evaluation of the role of the Universal Health Coverage Partnership in strengthening policy dialogue for health planning and financing: A protocol

Full name, postal address, e-mail and telephone number of the corresponding author

Full name: Emilie Robert

Postal address: Institut de recherche du Centre universitaire de santé McGill

2155, rue Guy, 5e étage

Montréal (QC)

Canada H3H 2R9

E-mail: emilie.robert2@mail.mcgill.ca

Telephone number: +1.514.998.9892

Full name, department, institution, city and country of all co-authors

- Emilie Robert

  Research Institute of the McGill University Health Centre (RI-MUHC) (at the time of submission)
  Montreal, Canada

  AND

  Transcultural Research and Intervention Team
  SHERPA (Recherche. Immigration. Société)
  CSSS de la Montagne
  Montreal, Canada

  AND

  Initiative collective sur l'approche réaliste pour l'évaluation et la synthèse des connaissances (ICARES)
  Montreal, Canada

- Valéry Ridde

  French Institute For Research on sustainable Development

For peer review only

For peer review only - http://bmjopen.bmj.com/site/about/guidelines.xhtml

CEPED (IRD-Université Paris Descartes), Universités Paris Sorbonne Cités, ERL INSERM SAGESUD
Paris, France

AND

University of Montreal Public Health Research Institute (IRSPUM)
Montreal, Canada

- Dheepa Rajan

Department of Health Systems Governance and Financing
World Health Organization
Geneva, Switzerland

- Omar Sam

Inter-Country Support Team
World Health Organization
Ouagadougou, Burkina Faso

- Mamadou Dravé

World Health Organization Office in Togo (at the time of the pilot mission)
Lomé, Togo

- Denis Porignon

Department of Health Systems Governance and Financing
World Health Organization
Geneva, Switzerland

Word count, excluding title page, abstract, references, figures and tables

4,057

For peer review only

ABSTRACT

Introduction

In 2011, the World Health Organization (WHO), the European Union and Luxembourg entered into a collaborative agreement to support policy dialogue for health planning and financing; these were acknowledged as core areas in need of targeted support in countries' quest towards universal health coverage (UHC). Entitled 'Universal Health Coverage Partnership', this intervention is intended to strengthen countries' capacity to develop, negotiate, implement, monitor, and evaluate robust and integrated national health policies oriented towards UHC. It is a complex intervention involving a multitude of actors working on a significant number of remarkably diverse activities in different countries.

Methods and analysis

The researchers will conduct a realist evaluation to answer the following question: How, in what contexts, and triggering what mechanisms, does the Partnership support policy dialogue for health planning and financing towards UHC? A qualitative multiple case study will be undertaken in Togo, Liberia, Democratic Republic of Congo, Cape Verde, Burkina Faso and Niger. Three steps will be implemented: 1) formulating Context-Mechanism-Outcome explanatory propositions to guide data collection, based on expert knowledge and theoretical literature; 2) collecting empirical data through semi-structured interviews with key informants and observations of key events, and analyzing data; 3) specifying the intervention theory.

Ethics and dissemination

The primary target audiences are WHO and its partner countries; international and national stakeholders involved in or supporting policy dialogues in the health sector, especially in low-income countries; and researchers with interest in UHC, policy dialogue, evaluation research, and/or realist evaluation. WHO-AFRO ethics review committee approved the study (AFR/ERC/2017/02.08). WHO as the funder of this research will not have any role in data collection and data analysis. However, any publication will require WHO approval.

KEY WORDS

Heath policy; realist evaluation; policy dialogue; universal health coverage; health planning; health financing

For peer review only

For peer review only - http://bmjopen.bmj.com/site/about/guidelines.xhtml

STRENGTHS AND LIMITATIONS OF THIS STUDY

1. The in-depth study of six countries ensures internal validity; and the potential to generalize the findings is increased by building explanations, taking the context into account in the production of outcomes, and using existing literature to provide theoretical foundations to the findings.
2. Methodological developments are expected, as the study is among the few realist evaluations to focus on policy dialogue, especially in low- and middle-income countries.
3. The study involves training and ongoing supervision of West African researchers who come from a different educational background, contributing to capacity building in health policy and systems research in low- and middle-income countries.
4. The study does not include countries or cases outside of the West African region.
5. Comprehensive data collection may be challenging in some countries with an unstable political situation.

For peer review only

For peer review only - http://bmjopen.bmj.com/site/about/guidelines.xhtml

INTRODUCTION

Universal health coverage (UHC) as a core objective within the Sustainable Development Goals is a journey in which multiple stakeholders, from the local, national and international levels, partake. It involves on-going discussions and negotiations among these stakeholders on the different facets of UHC, among which financing and planning questions.[1,2] Under the leadership of national health authorities, stakeholders will thus have to agree on priorities for action, and health financing and health system organization modalities.

Policy dialogue is where such discussions take place. The World Health Organization (WHO) describes policy dialogue as an iterative process that targets both the technical and policy aspects of the problem being discussed, involving evidence and sensitive policy discussions, in which a wide range of stakeholders participate.[3] This dialogue has a concrete objective, such as the development of a plan, a strategy or a policy. Policy dialogue is thus understood as a deliberative process by which different stakeholders are brought together to discuss issues of public policy to feed into decision-making.

In low- and middle-income country (LMIC) settings, the topic area of policy dialogue is attracting interest as a target intervention for programmes. Policy dialogue is seen as a key governance tool to ensure the coordination and alignment of stakeholders and to develop robust and comprehensive health policies anchored in the UHC concept.[4] Negotiations in the health sector may indeed be challenging due to the heavy influence external aid can have on national priorities, combined with the often limited government steering capacity to counter-balance that influence.[5,6] In the context of UHC, Ministries of Health's convening and brokering role takes on an added layer of importance, as they need to bring together a diverse range of interests to mobilize resources, prioritize interventions, and strengthen institutions for UHC.

Following this trend, policy dialogue is becoming a popular research object.[7] In the field of health policy and systems research in LMICs, scholars study policy dialogue as a knowledge translation instrument,[8-11] or as a decision-making process. In this case, they notably study the perceptions of what policy dialogue is.[12-14] The actual process of policy dialogue is more rarely investigated.[15,16] In high-income countries, research on policy dialogue as part of decision-making is more diffuse and includes research on research-policy dialogue,[17,18] stakeholder dialogue,[19,20] or social dialogue.[21]

In 2011, WHO, the European Union and Luxembourg entered into a collaborative agreement called the *Universal Health Coverage Partnership* to support policy dialogue for health planning and financing as core topic areas of UHC. This Partnership also responds to a resolution from the 64th World Health Assembly calling on WHO to support countries in developing more robust and evidence-based health policies and plans based on an inclusive and participatory process.[22] Implemented in 28 countries, the Partnership is a complex intervention involving a multitude of actors working on a significant number of very diverse activities in different countries. Moreover, it takes place in the policy arena where policy-making is complex and dynamic. This study aims to examine the Partnership through a realist lens, to better understand how and in what contexts it can, through WHO in-country support, contribute to strengthening policy dialogue.

For peer review only - http://bmjopen.bmj.com/site/about/guidelines.xhtml

REALIST APPROACH

Traditional evaluation methods face three limitations when studying complex interventions in dynamic contexts, as is the case of the Partnership. First, establishing a causal relationship between the Partnership and its expected outcomes in terms of policy formulation and donors alignment on one hand, and between the Partnership and the indicators usually used in classical evaluations on the other, is onerous as such relationships are not direct and are strongly colored by the contexts within which WHO intervenes. Second, WHO's role in the Partnership complements its traditional role as technical advisor. Demonstrating how this new approach helps support policy dialogue, and painting a clear picture of the challenges involved, is difficult to do using traditional evaluation methods. Finally, the Partnership has been implemented in several countries representing a wide variety of contexts, which raises the third limitation of traditional evaluations: the inability to take context into account in explaining an intervention's outcomes and to draw parallels between the experiences of different countries. Therefore, the realist approach was deemed most appropriate as a frame to evaluate the UHC Partnership.

A realist evaluation entails the premise that an intervention does not function on its own and is not, in itself, what produces an outcome.[23] Instead, an outcome is the product of the interaction between a mechanism triggered by the intervention and the context in which the intervention is implemented. A mechanism should be understood as the way in which the actors involved in an intervention react to and reason about the resources made available to them as part of the intervention.[24]

A realist evaluation, therefore, does not attempt to control the variables associated with the implementation context. Rather, it seeks to observe, in the natural environment, the regular—although not systematic—occurrence of an outcome in a particular context, when a mechanism is triggered. These iterations of interactions are referred to as context–mechanism–outcome (CMO) configurations. The objective of a realist evaluation is thus to identify, in the empirical data, iterative interactions between an intervention's context, mechanisms, and outcomes. Operationally, it begins with a reflection on the intervention theory and results in the production of an explanatory theory, which is sufficiently abstract to explain how the intervention functions in different contexts. This anchoring in theory requires an explicitly iterative process between the conceptual and theoretical literature underlying the rationale behind the intervention, and the empirical data.

INTERVENTION THEORY

Several sources of information were used to develop an initial intervention theory of the Partnership. In addition to reviewing the documents produced by WHO, several meetings were held with stakeholders from WHO headquarters' Department of Health Systems Governance and Financing which implements the Partnership. These meetings aimed to make explicit the logical reasoning underlying the need for and the rationale behind the Partnership. Observations of two annual inter-country meetings bringing together partner countries and WHO teams were also conducted. This process resulted in a generic intervention theory that was discussed at a workshop in Burkina Faso in 2017 and subsequently adjusted to take into account the participants' input (Figure 1).

For peer review only - http://bmjopen.bmj.com/site/about/guidelines.xhtml

In the frame of the Partnership, WHO supports policy dialogue processes for health planning and health financing. According to WHO, such processes should be inclusive and participatory, fed by knowledge and evidence, and led by the Ministries of Health (MoH). WHO's role is to support MoH to lead structured and transparent policy dialogues. The ultimate goal is to contribute to strengthening health systems, especially its governance function, with the assumption that health systems strengthening is the principal means to achieve UHC. WHO support through the Partnership manifests itself through a hard component, including financial resources for a set of activities as well as human resources, and a soft component, which refers to a specific approach to supporting the policy dialogue. The hard component of WHO support includes strengthening of the capacities of MoH to conduct policy dialogue processes. This involves WHO country office on-going support as well as training. It also involves strengthening of the quality of the policy dialogue itself with the generation of relevant evidence, data and normative guidelines, as well as *ad hoc* expertise. With regard to WHO's approach, three characteristics are essential: flexibility, i.e. the ability to adapt to the changing priorities of the MoH; responsiveness, or the ability to position and respond to requests from the MoH; and proactivity, i.e. the ability to anticipate implementation steps and expectations or needs of the MoH.

The resulting policy dialogue is expected to enable the formulation of robust and comprehensive health policies, strategies and plans that promote the principles of UHC such as equity, financial risk protection, and quality of care. It should also facilitate the alignment of multiple stakeholders, be they donors, civil society, or related ministries. Thus, the Partnership is supposed to contribute to upholding the principles of the Paris Declaration to ensure effective development cooperation. Finally, supporting MoH in the organization of such dialogues is expected to strengthen the capacities and leadership position of the MoH. To summarize, WHO has three principal roles within the Partnership: convener of policy dialogue processes, policy broker, and a relatively traditional role of advisor to the MoH.

(Insert Figure 1)

WHO may act as a "boundary spanner", i.e., an actor who brings together partners with common interests and goals (such as UHC), while striving to establish a climate of trust, optimism, and perseverance.[25] The concept of policy broker is similar to that of boundary spanner. The role of a broker is to bring together groups of actors whose interests and beliefs differ about the issue of concern in the policy dialogue.[26] The broker's objective is to promote consensus in a climate of trust, to stabilize the policy dialogue and to find workable compromises. However, his role is not necessarily a disinterested one, and he might be pursuing a strategic objective, which would bring him closer to the role of a policy entrepreneur. A policy entrepreneur has an explicit strategic objective, and proposes ideas and stimulates opportunities in ways that are intended to create a demand for the solution he is proposing.[26] These roles are, however, ideal types positioned at two ends of a continuum.[26] Depending upon the contexts in which it intervenes, the processes in which it is involved, and the issues being discussed, WHO may be called upon to play one or another of these various roles.

For peer review only - http://bmjopen.bmj.com/site/about/guidelines.xhtml

METHODS AND ANALYSIS

RESEARCH QUESTIONS

The question that will guide the study is: How, in what contexts, and triggering what mechanisms does the Partnership support policy dialogue for health planning and health financing towards UHC, and with what outcomes?

Four specific objectives have been established to respond to this research question:

1) To highlight the contexts in which the Partnership, through WHO support, can, or cannot:

- act as a broker, promoting the dissemination and use of data and evidence in policy dialogue and seeking workable compromises;

- act as a convener, creating synergy among the actors involved in policy dialogue for health planning and financing;

- play its role as an advisor, ensuring transparent and structured policy dialogue in accordance with available evidence and equity principles; and supporting MoH in its leadership and stewardship functions.

2) To identify the mechanisms at work and the outcomes—direct or indirect, expected or not—produced;

3) To clarify the process chains and the links between them, especially highlighting feedback loops;

4) To specify how, in what contexts, triggering what mechanisms, and with what outcomes the Partnership, through WHO support, enhances policy dialogue for health planning and financing towards UHC.

STUDY DESIGN

A realist multiple case study will be undertaken.[27] Table 1 presents a summary of the proposed design. The countries in which the study will be conducted are Togo, Liberia, Democratic Republic of Congo, Cape Verde, Burkina Faso and Niger. Due to feasibility concerns, only partner countries of the African continent were selected. They were sampled using a non-probabilistic approach. The objective was to select cases—or countries—purposively, so as to obtain a diversity and depth of information on the Partnership. This is therefore a contrasted sample. When selecting cases, purposive sampling involves establishing selection criteria that ensure contrasting cases. The criteria are based on theoretical assumptions about the influence they may have on the Partnership and policy dialogue. An initial list was shared with WHO in order to select the criteria that could be informed by their experience. Based on their country knowledge and experience, they then suggested countries matching the criteria, based on their country knowledge and experience (Table 2).

For peer review only - http://bmjopen.bmj.com/site/about/guidelines.xhtml

Table 1. Summary of the study design

**Epistemological foundation**	Critical realism
**Design**	Multiple case studies
**Cases**	Countries involved in the Partnership
**Units of analysis**	Policy dialogue processes supported by WHO as part of the country roadmaps
**Type of sample**	Contrasted
**Case sampling strategy**	Purposive sampling

Each country has a roadmap for UHC Partnership work, in line with its needs. In this study, the unit of analysis is a policy dialogue process defined in the country's roadmap. One process (unit of analysis) will be observed in each country (case). Each process was selected in collaboration with the country teams, taking into account on-going support at the start of data collection, as well as opportunities for observation. In Togo and DRC, the processes under study are the policy dialogues around the national health financing strategy, whereas in Cape Verde, Niger and Togo, there are the policy dialogues for the elaboration of the national or regional health plans. In Liberia, the process under study is the elaboration of the IHP+ (International Health Partnership) Compact, which is an aid coordination agreement amongst health sector stakeholders. The process selected determines the research sub-questions to be addressed in each case, the documentation to be obtained, the informants to interview, and the topics to be covered in interviews.

For peer review only

For peer review only - http://bmjopen.bmj.com/site/about/guidelines.xhtml

Table 2. Case selection criteria and reason for sampling

Criteria*	Togo	Liberia	DRC	Cape Verde	Burkina Faso	Niger
Duration of WHO support	4	4	3	3	3	3
Political stability (0=instable; 4=stable)	3	3	2	3	3	1,5
Ministerial stability (0=instable; 4=stable)	3	3	3	2	2	1
Openness to policy dialogue (0=not open; 4=open)	3	3	3	2	2	1,5
Ministry of Health leadership capacity (0=weak; 4=strong)	3	3	3	3	3	1,5
Implementation of the national road map (0=weak; 4=good)	3	3	3	2	3	1
Presence of entry points	Yes	Yes	Yes	Yes	Yes	Yes
Additional observations		*Ebola epidemic*	*Devolution*	*Archipelago – Lower middle-income country*		

** At the time of case selection*
Note: Criteria were noted on a scale from 0 to 4, where 0 is the lowest and 4 the highest. For example, a score of 4 for Togo for criteria 'Duration of WHO support' means that the country benefited from WHO support for the longest period of time.

STUDY STEPS

Step 1: Formulating context-mechanism-outcome propositions

Within a realist perspective, researchers raise the questions of the mechanisms, that is, how actors react to the new resources made available through an intervention, and of the contexts that trigger or hinder these mechanisms. The initial intervention theory was divided into two sub-theories to clarify what mechanisms and contexts may interact (Figure 2 and Figure 3). Contexts and mechanisms derive from three sources: literature on partnership synergy;[25,28,29] a scoping study on policy dialogue,[30] looking into knowledge translation, policy change, and development studies literature; and interviews conducted with international experts involved in the Partnership. We describe below both sub-theories, highlighting mechanisms (M), outcomes (O), and potential contexts (C).

The first sub-theory focuses on the Partnership and its expected outcomes (Figure 2). It posits that capacity building, through training, technical expertise and on-going support from WHO, would empower MoH (M), while triggering a shared understanding of governance and policy dialogue (M). This should result in MoH leading inclusive and participatory policy dialogues (O). Furthermore, the on-going support provided to MoH, combined with WHO approach (i.e. proactiveness, responsiveness, flexibility), should prompt mutual trust (M), which in turn should strengthen their collaboration for policy dialogues (O). Finally, the sub-theory postulates that the

For peer review only

availability of evidence and data provided through the Partnership would bring about a shared understanding of the needs and policy options by MoH and WHO (M), contributing to an evidence-informed policy dialogue (O). In sub-theory 1, mechanisms may be triggered by the Partnership when: existing national governance arrangements facilitate the organization of policy dialogue (C); WHO and MoH have enduring relationships (C); they have the expected expertise and skills (C); they both endorse collaborative and cross-sectoral ways of working (C); human resources from both institutions who are involved in the Partnership are stable (C); and there is synergy between the Partnership and international stakeholders' priorities and plans to strengthen MoH leadership (C).

(Insert Figure 2)

The second sub-theory focuses on policy dialogue and its expected outcomes (Figure 3). It posits that an evidence-informed policy dialogue would first trigger a shared understanding among stakeholders of the challenges and possible ways of action (M). This should contribute to more robust and comprehensive health policies, strategies and plans (O) through the formulation of commonly agreed workable propositions. An inclusive and participatory policy dialogue should also prompt a shared understanding of stakes, values and interests of the various actors involved (M), as well as buy-in of the decisions (M), strengthening stakeholders' alignment on national health priorities (O). Finally, brought under the responsibility of MoH, it should foster MoH sense of ownership of the process (M), contributing to strengthening its leadership (O). Such an outcome may also be the consequence of stakeholders' trust in a structured and transparent policy dialogue process (M). In sub-theory 2, policy dialogue may trigger such mechanisms when: participants show openness to policy dialogue (C); they have the capacity and legitimacy to actively participate (C); they have previous positive experience of policy dialogue (C); their organizations' culture value collaboration and cross-sectoral ways of working (C); policy dialogue benefit from financial and technical support (e.g. the Partnership) (C); there is synergy between the global and national health agendas (C); and policy dialogue matches socio-cultural forms of communication and participation to governance and formal events (C).

(Insert Figure 3)

For each case, we will formulate at least one CMO proposition for each outcome, based on these theories. Additional theoretical literature may be used to fill in potential knowledge gaps, such as capacity building literature and theories about organizational change.

Step 2: Collecting and analyzing empirical data

Once the propositions are developed, an interview guide will be drafted for each case. The interviewee is there to help the researcher 'refine' the theory, as each interviewee holds a piece of the complex puzzle of the intervention.[23] Consequently, the interview guide will be adjusted to the interviewee. For example, it is expected that WHO informants will be knowledgeable about the Partnership and its outcomes (sub-theory 1), while other stakeholders will be able to share their experience as policy dialogue participants (sub-theory 2).

Three sources of information will be used. First, semi-structured interviews will be conducted with key informants who will be jointly identified by the researchers and WHO. An initial list

For peer review only - http://bmjopen.bmj.com/site/about/guidelines.xhtml

will be compiled by WHO and then supplemented using a snowballing technique. The aim is to meet with as many as possible of the participants of the policy dialogue and avoid investigating merely the positive side of the coin, and thus possible bias. Depending on informants' availability, interviews will be conducted in person or by telephone. The interviews will be audio-recorded, except when not allowed by the participants. They will also be recorded in notes taken systematically by the researchers. Recordings will subsequently be transcribed. Between 20 and 30 informants will be interviewed for each case. The principle of empirical saturation will be applied to determine the best time to bring the data collection to a close.[31]

Observations will also be conducted of activities organized either directly by WHO or with WHO collaboration within the Partnership context. These observations will be described and recorded in the logbooks of researchers. Also, all documentation related to the Partnership (e.g. national roadmaps, project documents, annual reports) will be reviewed.

Furthermore, to gain a deeper understanding of the national health policy contexts, and particularly of the interests, power relationships, and positioning of the actors involved in policy dialogue, a stakeholder analysis will be performed for each case.[32] First, we will compile a list of stakeholders involved in policy dialogue for each case. Then, for each stakeholder, we will attempt to describe their power of action and influence, as well as their interest and involvement with respect to UHC and policy dialogue. The last step will be to analyze how these stakeholders react to the roles played by WHO in policy dialogue, and how their reactions influence these two processes. This exercise will be conducted before the interviews in collaboration with the WHO teams, who have first-hand knowledge of the field. The analysis will be completed during the interviews with the stakeholders included on the list.

Using an iterative approach during data collection, we will deepen our understanding of the intervention theory, including "hypotheses about their subjects' reasoning within a wider model of their causes and consequences" (p.163).[23] Data analysis will contribute to deepening our understanding further. It will start with a thorough description of the barriers and facilitators for each piece of the intervention theory. We will then move to identify in the data the interaction between context, mechanisms, and outcomes. Finally, we will attempt to identify - across interviews - patterns of such interactions, called context-mechanism-outcome (CMO) configurations. Both barriers and facilitators and CMO configurations will be identified in the data through content analysis performed using NVivo software. For the latter, we will: 1) identify the outcomes (description); 2) determine the contextual components related to the outcomes (resolution); 3) reconstruct the links between context and outcomes theoretically (abduction); 4) identify the mechanisms (retroduction).[33] Retroduction aims to construct the theoretical explanation of a phenomenon.[34,35] More specifically, this retrospective reasoning allows moving from describing the outcome to describing what produced that outcome and the context within which it takes place,[36,37] via the mechanisms.[38] In case of missing pieces of evidence, additional interviews will be conducted whenever possible.

Step 3: Specifying the theory

In order to specify the intervention theory across cases, a transversal analysis will be conducted using C-M-O configurations as primary data. Such configurations may be incomplete, with the mechanism generally not being explicit. The missing links will thus be inferred from the

For peer review only - http://bmjopen.bmj.com/site/about/guidelines.xhtml

theoretical literature, which provides clues to explain the links between causes and outcomes, following a process of intellectual reasoning based on two questions: How can we explain the mechanism in this context and considering this outcome? Is this interpretation supported by the theoretical literature?

This inference takes the form of a demi-regularity when the configuration re-appears several times. A demi-regularity is a regular—although not systematic—occurrence in the interactions among the context, the mechanism(s), and the outcomes.[39] When a configuration is identified only once but its interpretation is supported by the theoretical literature, it is also taken into account, on the condition that it helps to clarify how and under what circumstances the Partnership contributes to each outcome. Each demi-regularity will provide one or more piece of information that, when combined, present a more complete and precise picture of the Partnership. Such an analysis will contribute to specify the explanatory realist theory of the Partnership. Table 3 summarizes the study process, including sources of data, methods and status. The research should be completed by mid-2019.

Table 3. Summary of the study process

Steps	Data sources	Methods	Status
Building a generic intervention theory	▪ Program documents<br>▪ Meetings with stakeholders from WHO headquarters' Department of Health Systems Governance and Financing<br>▪ Observations of annual inter-country meetings (n=2)	Iterative co-building process	Completed (2016)
Formulating CMO explanatory propositions	▪ Literature review on policy dialogue<br>▪ Semi-structured interviews with experts at WHO headquarters and at regional level (n=13)	▪ Scoping study<br>▪ Content analysis	On-going (2017-18)
Identifying CMO configurations	▪ Semi-structured interviews with WHO national experts, MoH counterparts, participants of policy dialogues (e.g. civil society, international stakeholders, connected Ministries and public institutions) in each country<br>▪ Observations of policy dialogues	▪ Stakeholders analysis<br>▪ Content analysis	2018
Specifying intervention theory	▪ CMO configurations from cases	Transversal analysis	2019

Patients and public involvement

There is no patient or public involvement in this study.

ETHICS AND DISSEMINATION

Protection of key informants

For peer review only - http://bmjopen.bmj.com/site/about/guidelines.xhtml

The research objectives and a consent form will be sent by email to informants before the interviews. The rights of informants include the right to refuse to participate in the interview, to end their participation in the study at any time, and to not allow their statements to be audio-recorded. Informed consent will be sought in writing from informants. We expect that all informants will be literate, given that they occupy positions of responsibility.

Informants' statements will be kept confidential. The data collected will be kept anonymous at the time of transcription, i.e., informants' names will be concealed, as well as their title and their function. Transcriptionists and everyone involved in data collection and analysis will sign confidentiality agreements. A copy of the data will be maintained on a password-protected virtual platform. The data will be destroyed 18 months after the signing of the consent forms.

ETHICS APPROVAL OF RESEARCH

The research protocol has been approved by the Ethics Committee of the WHO Regional office for Africa (ARF/ERC/2017/02.08), as well as the Ethics Committee of each country included in the study.

IMPORTANCE OF THE STUDY

First, in providing lessons on how the Partnership works and what proximal and unexpected outcomes it produces, the research should allow WHO and Partnership donors to improve the Partnership content, to adapt it better to the contexts in which it is implemented, and to tailor the nature of the policy dialogue support to take into account the diverse influences that foster or undermine productive collaboration among stakeholders. This more constructive collaboration should in turn contribute to more robust and actionable health policies, as a means to strengthen health systems so that they can deliver the quality and affordable health services users are entitled to under the banner of UHC.

Second, the realist evaluation has been little used to study interventions on an international scale. Thus, this study will be a unique example in health policy and systems research, and will offer important methodological lessons. Furthermore, it entails the training and ongoing supervision of a team of West African researchers coming from various educational backgrounds and with different expertise. This project will help strengthen capacities in health policy and systems research in low- and middle-income countries.

Finally, in terms of the topics addressed, the nature of the knowledge produced will be unique and will provide a deeper understanding of the issues associated with interventions aimed to support policy dialogue. As such, it adds to the findings on policy dialogue reported in the *BMC Health Services Research* special issue.[40]

DISSEMINATION OF RESULTS

The results of the study will be disseminated on several levels: WHO, including the headquarters, the regional teams, and the country teams; WHO's national and international partners, particularly MoH and donors; and the scientific community. With regards to the scientific community, empirical findings and methodological issues will be shared in related

For peer review only - http://bmjopen.bmj.com/site/about/guidelines.xhtml

symposia and in peer-reviewed journals. Any intention to publish the findings must be approved by WHO before submitting to the journal.

For peer review only

For peer review only - http://bmjopen.bmj.com/site/about/guidelines.xhtml

REFERENCES

1. Schmets G, Rajan D, Kadandale S. Strategizing national health in the 21st century: a handbook. Geneva: World Health Organization, 2016.

2. Evans T, Pablos-Mendez A. Shaping of a new era for health financing. *Lancet* 2016;**387**(10037):2482-4. doi: 10.1016/S0140-6736(16)30238-0

3. World Health Organization. *Health Systems Strengthening: Glossary*. Geneva, Switzerland: World Health Organization, 2011.

4. Rajan D, Adam T, El Husseiny D, et al. *Briefing Note - Policy dialogue: What it is and how it can contribute to evidence-informed decision-making*. Geneva: WHO, 2015.

5. Uneke CJ, Ezeoha AE, Ndukwe CD, et al. Enhancing leadership and governance competencies to strengthen health systems in Nigeria: assessment of organizational human resources development. *Healthc Policy* 2012;**7**(3):73-84.

6. Quinn M. *Governance and Health in Post-Conflict Countries: The Ebola Outbreak in Liberia and Sierra Leone*. New York: International Peace Institute, 2016.

7. Nabyonga-Orem J, Ota M, Mwisongo A, et al. Health policy dialogue: lessons from Africa. *BMC Health Serv Res* 2016;**16**(Supplement 4)

8 Lavis JN, Boyko J, Oxman AD, et al. SUPPORT Tools for evidence-informed health Policymaking (STP) 14: Organising and using policy dialogues to support evidence-informed policymaking. *Health Res Policy Syst* 2010;**10**(5):514-19 doi: 10.1186/1478-4505-7-S1-S14.

9. Uneke CJ, Ebeh Ezeoha A, Uro-Chukwu H, et al. Promoting Evidence to Policy Link on the Control of Infectious Diseases of Poverty in Nigeria: Outcome of A Multi-Stakeholders Policy Dialogue. *Health Promot Perspect* 2015;**5**(2):104-15. doi: 10.15171/hpp.2015.013

10. Drechsel P, Cofie OO, Van Veenhuizen R, et al. Linking research, capacity building, and policy dialogue in support of informal irrigation in urban West Africa. *Irrig Drain* 2008;**57**(3):268-78. doi: 10.1002/ird.430

11. Babu SC, Ergeneman A. Policy Dialogue with Indian Parliamentarians: Outcomes and Lessons from the CGIAR. *J Int Commun* 2005;**11**(1):41-54.

12. Nabyonga-Orem J, Dovlo D, Kwamie A, et al. Policy dialogue to improve health outcomes in low income countries: What are the issues and way forward? *BMC Health Serv Res* 2016;**16** doi: 10.1186/s12913-016-1450-2

13. Kwamie A, Nabyonga-Orem J. Improved harmonisation from policy dialogue? Realist perspectives from Guinea and Chad. *BMC Health Serv Res* 2016;**16** doi: 10.1186/s12913-016-1458-7

For peer review only - http://bmjopen.bmj.com/site/about/guidelines.xhtml

14. Molenaers N. The Great Divide? Donor perceptions of budget support, eligibility and policy dialogue. *Third World Q* 2012;**33**(5):791-806. doi: 10.1080/01436597.2012.677311

15. Ade N, Réne A, Khalifa M, et al. Coordination of the health policy dialogue process in Guinea: Pre- and post-Ebola. *BMC Health Serv Res* 2016;**16** doi: 10.1186/s12913-016-1457-8

16. White LG. Policy Reforms in Sub-Saharan Africa - Conditions for Establishing a Dialogue. *Stud Comp Int Dev* 1990;**25**(2):24-42.

17. Scholten P, Verbeek S. Politicization and expertise: Changing research-policy dialogues on migrant integration in Europe. *Sci Public Policy* 2015;**42**(2):188-200. doi: 10.1093/scipol/scu040

18. Lavis JN, Boyko JA, Gauvin FP. Evaluating deliberative dialogues focussed on healthy public policy. *BMC Public Health* 2014;**14**

19. Baumann C, White S. Collaborative Stakeholder Dialogue: A Catalyst for Better Transport Policy Choices. *Int J Sustain Transp* 2015;**9**(1):30-38.

20. Susskind L, Hoben M. Making regional policy dialogues work: A credo for metro-scale consensus building. *Temp Envtl Law & Tech J* 2004:123-38.

21. Perin E. Au coeur du dialogue social sectoriel européen : entre coordination européenne et négociations nationales : analyse de la mise en oeuvre des textes de nouvelle génération. Université catholique de Louvain, 2014.

22. Executive Board of the World Health Assembly. *Strengthening national policy dialogue to build more robust health policies, strategies and plans*. In: Executive Board of the World Health Assembly, ed. EB128R12, 2011.

23. Pawson R, Tilley N. Realistic evaluation. London, United Kingdom: Sage Publications, 1997.

24. Lacouture A, Breton E, Guichard A, et al. The concept of mechanism from a realist approach: a scoping review to facilitate its operationalization in public health program evaluation. *Implement Sci* 2015;**10**(1):153. doi: 10.1186/s13012-015-0345-7

25. Jones J, Barry MM. Exploring the relationship between synergy and partnership functioning factors in health promotion partnerships. *Health Promot Int* 2011;**26**(4):408-20. doi: 10.1093/heapro/dar002

26. Christopoulos D, Ingold K. Exceptional or just well connected? Political entrepreneurs and brokers in policy making. *Eur Political Sci Rev* 2015;**7**(3):475-98. doi: 10.1017/S1755773914000277

27. Yin RK. Case study research: Design and methods (Fifth edition). Thousand Oaks, CA: Sage, 2014.

For peer review only

For peer review only - http://bmjopen.bmj.com/site/about/guidelines.xhtml

28. Lasker RD, Weiss ES, Miller R. Partnership synergy: a practical framework for studying and strengthening the collaborative advantage. *Milbank Q* 2001;**79**(2):179-205, III-IV.

29. Jagosh J, Macaulay AC, Pluye P, et al. Uncovering the Benefits of Participatory Research: Implications of a Realist Review for Health Research and Practice. *Milbank Q* 2012;**90**(2):311-46.

30. Robert E, Ridde V. « Policy dialogue » (dialogue sur les politiques) : une synthèse exploratoire des écrits scientifiques. *Cahiers REALISME* 2018;**(sous presse)**(16)

31. Pires A. Echantillonnage et recherche qualitative : essai théorique et méthodologique. In: Poupart J, Deslauriers J-P, Groulx L-H, et al., eds. La recherche qualitative: Enjeux épistémologiques et méthodologiques. Montréal, QC: Gaëtan Morin 1997:113-69.

32. Varvasovszky Z, Brugha R. Stakeholder analysis: A review. *Health Policy Plan* 2000;**15**(3):239-46. doi: 10.1093/heapol/15.3.239

33. Abimbola S, Molemodile SK, Okonkwo OA, et al. 'The government cannot do it all alone': realist analysis of the minutes of community health committee meetings in Nigeria. *Health Policy Plan* 2016;**31**(3):332-45. doi: 10.1093/heapol/czv066

34. Bhaskar R. Reclaiming reality: A critical introduction to contemporary philosophy. London: Verso, 1989.

35. Trimarchi M. *Theory building: A realist methodology for case study driven research*. Faculty of Business Working Paper Series. Sippy Downs, Australia: University of the Sunshine Coast, 1998:19-35.

36. Bhaskar R. Scientific realism and human emancipation. London, United Kingdom: Verso, 1986.

37. Wai-chung Yeung H. Critical realism and realist research in human geography: A method or a philosophy in search of a method? *Prog Hum Geogr* 1997;**21**(1):51-74.

38. McEvoy P, Richard D. A critical realist rationale for using a combination of quantitative and qualitative methods. *J Res Nurs* 2006;**11**(1):66-78. doi: 10.1177/1744987106060192

39. Pawson R. Evidence-based policy: A realist perspective. London, United Kingdom: Sage Publications, 2006.

40. Nabyonga-Orem J, Ousman K, Estrelli Y, et al. Perspectives on health policy dialogue: Definition, perceived importance and coordination. *BMC Health Serv Res* 2016;**16** doi: 10.1186/s12913-016-1451-1

For peer review only - http://bmjopen.bmj.com/site/about/guidelines.xhtml

LEGEND

Figure 1. Generic intervention theory of the UHC Partnership

Figure 2. Initial sub-theory of UHC Partnership (sub-theory 1)

Figure 3. Initial sub-theory of policy dialogue (sub-theory 2)

For peer review only

For peer review only - http://bmjopen.bmj.com/site/about/guidelines.xhtml

AUTHORS' CONTRIBUTIONS

ER and VR designed the study. DR, OS, MD, and DP provided critical data and information regarding the intervention, the sampling of cases and feasibility of the research. ER drafted the first version of the protocol. All authors critically revised the study protocol for important intellectual content and approved the manuscript.

FUNDING STATEMENT

This work is funded by the Department of Health Systems Governance and Financing of the World Health Organization.

COMPETING INTERESTS STATEMENT

ER and VR are funded as research consultants by the Department of Health Systems Governance and Financing of the World Health Organization. DR, OS, MD and DP are experts at the World Health Organization, and involved within the Universal Health Coverage Partnership.

ACKNOWLEDGEMENTS

We would like to thank the following collaborators at WHO: Juliet Nabyonga, Martin Ota, and Kira Koch. We would also like to express our gratitude to the WHO country office and Sébastien Segniagbeto in Togo for their support during the pilot mission, as well as researchers in countries for support with national ethics committees. We finally would like to acknowledge Donna Riley for the translation.

For peer review only

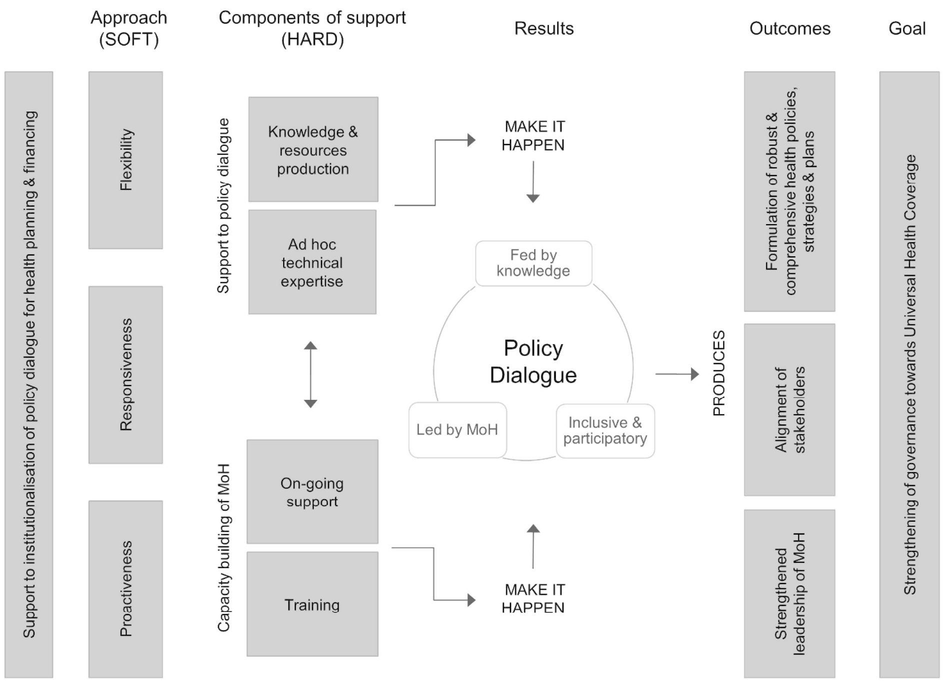

Caption : Generic intervention theory of the UHC Partnership

254x190mm (300 x 300 DPI)

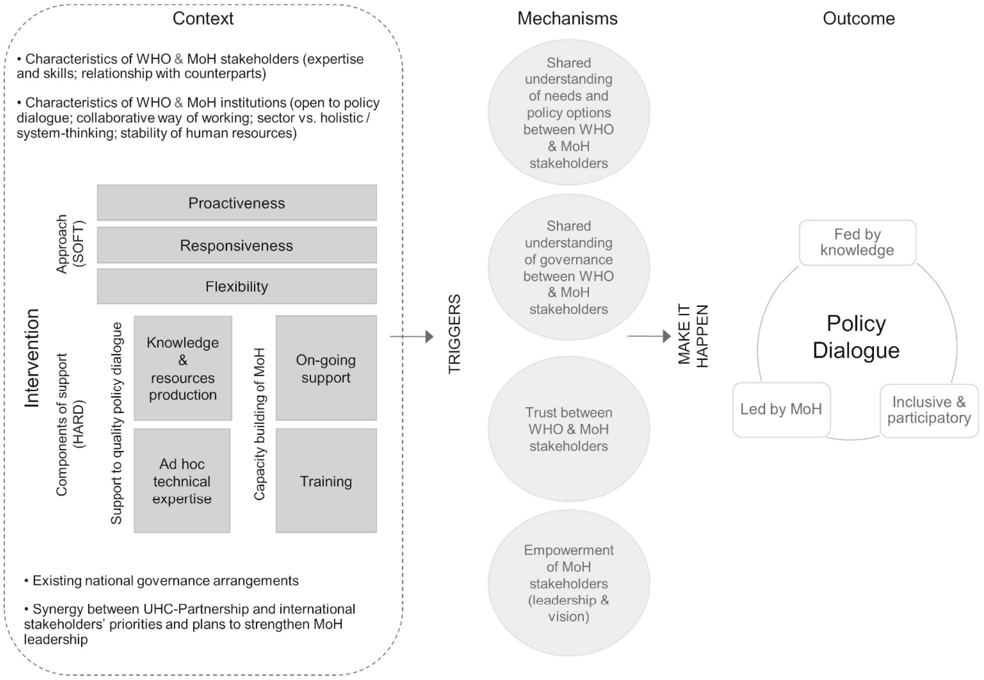

Caption : Initial sub-theory of UHC Partnership (sub-theory 1)

254x190mm (300 x 300 DPI)

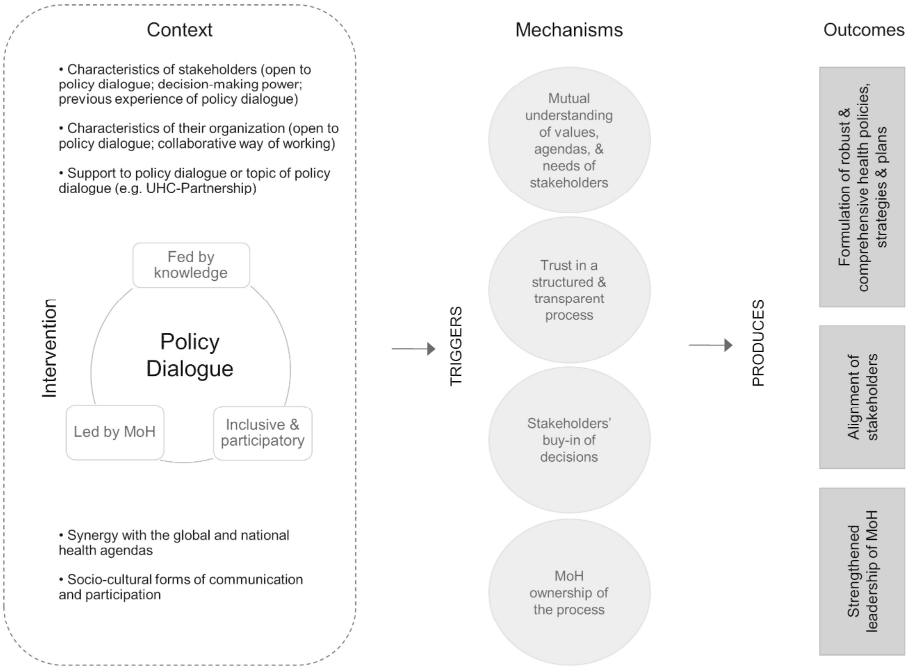

Caption : Initial sub-theory of policy dialogue (sub-theory 2)

254x190mm (300 x 300 DPI)
